# Neutrophil extracellular traps promote invasion and metastasis via NLRP3-mediated oral squamous cell carcinoma pyroptosis inhibition

**DOI:** 10.1038/s41420-024-01982-9

**Published:** 2024-05-02

**Authors:** Rundong Zhai, Zizhen Gong, Mengqi Wang, Zihui Ni, Jiayi Zhang, Mengyao Wang, Yu Zhang, Fanrui Zeng, Ziyue Gu, Xingyu Chen, Xiudi Wang, Pengcheng Zhou, Laikui Liu, Weiwen Zhu

**Affiliations:** 1 State Key Laboratory Cultivation Base of Research, Prevention and Treatment for Oral Diseases, Nanjing Medical University, Jiangsu, China; 2 Department of Basic Science of Stomatology, The Affiliated Stomatological Hospital of Nanjing Medical University, Jiangsu, China; 3 Jiangsu Province Engineering Research Center of Stomatological Translational Medicine, Jiangsu, China; 4 Department of Oral and Maxillofacial-Head Neck Oncology, Shanghai Ninth People’s Hospital, Shanghai Jiao Tong University School of Medicine, Shanghai, 200011 China; 5 College of Stomatology, Shanghai Jiao Tong University, National Center for Stomatology, National Clinical Research Center for Oral Diseases, Shanghai Key Laboratory of Stomatology, Shanghai, 200011 China

**Keywords:** Oral cancer, Cancer microenvironment

## Abstract

Neutrophil extracellular traps (NETs) are reticular structures composed of neutrophil elastase (NE), cathepsin G (CG) and DNA-histone enzyme complexes. Accumulating evidence has revealed that NETs play important roles in tumor progression, metastasis, and thrombosis. However, our understanding of its clinical value and mechanism of action in oral squamous cell carcinoma (OSCC) is limited and has not yet been systematically described. Here, we aimed to investigate the clinical significance of NETs in OSCC and the mechanisms by which they affect its invasive and metastatic capacity. Our results demonstrated that high enrichment of NETs is associated with poor prognosis in OSCC, and mechanistic studies have shown that NE in NETs promotes invasion and metastasis via NLRP3-mediated inhibition of pyroptosis in OSCC. These findings may provide a new therapeutic approach for OSCC.

## Introduction

Oral squamous cell carcinoma (OSCC) is the most common malignancy of the oromaxillofacial region, accounting for more than 90% of oral cancers [1]. Although therapeutic strategies have greatly improved in recent years, the 5-year survival rate of OSCC remains at approximately 60% [2–4], with cancer metastasis or recurrence being the main cause of death [5]. Therefore, the identification of novel therapeutic targets that focus on the pathogenesis of metastasis and recurrence is needed to develop a more effective treatment for OSCC.

Recent publications have confirmed that the invasion and metastasis of tumors are related to the characteristics of the tumor cells themselves, and are closely related to their local environment. The tumor microenvironment (TME) includes various cellular (e.g., endothelial cells, fibroblasts, and immune cells,) and extracellular components (e.g., cytokines, growth factors, hormones, and extracellular matrix) that surround the tumor cells and are nourished by vascular networks. The TME plays a key role in the metabolism, proliferation, invasion, and metastasis of tumors, and has a profound impact on therapeutic efficacy [6–9]. However, the underlying mechanism remains unclear. Therefore, the aim of radical treatment of OSCC is to clarify the key mechanism between the TME and its invasion and metastasis.

Neutrophils, the most abundant leukocytes in the circulatory system, are important components of the TME. They play an important role in the connection between cancer and inflammation as well as in tumor progression and metastasis [10]. After recruitment to the TME, neutrophils release cytokines and enzyme-like substances that affect the recruitment and activation of inflammatory cells in the TME [11]. However, their roles in tumor biology, including tumor progression and invasion, remain controversial. Numerous studies have suggested that neutrophils have a tumor-promoting role in cancer progression [12, 13]. On the contrary, several other studies have shown that neutrophils can exert antitumor effects by activating an immune response against tumors and promoting the clearance of tumor cells [14].

In 2004, an extracellular pathogen-killing mode of neutrophils, called neutrophil extracellular traps (NETs), was first proposed by Brinkmann and Zychlinsky [15]. Unlike neutrophil apoptosis and necrosis, the formation of NETs are a novel cell death program [16]. During this process, the nuclear membrane of activated neutrophils dissolves, followed by the disintegration of the plasma membrane, release of nuclear contents, decondensation of chromatin, and release of chromosomes containing granule proteins into the extracellular space [17]. The contents of the secretory granules of neutrophils, including neutrophil elastase (NE), matrix metalloproteinase 9 (MMP9), and cathepsin G (CG), are physically associated with chromatin [15].

NETs are important components of the innate immune response and are associated with multiple diseases [18–20]. Numerous studies have indicated that NETs form in the TME and are involved in tumor development, with their dual roles in tumor immune editing, tumor progression, tumor metastasis, and tumor-associated thrombosis [14, 21]. In contrast, NETs can exert anti-tumor effects. The components of NETs, such as myeloperoxidase (MPO) and histones, can eliminate tumors and inhibit tumor progression and metastasis [22, 23]. However, in some malignancies, NETs exhibit tumor-promoting activity. NET proteases can degrade the extracellular matrix to promote cancer cell invasion and metastasis [24, 25]. NETs can trap and serve as substrates for cancer cell adhesion, thereby facilitating metastasis [26, 27]. Moreover, circulating NETs are thought to cause organ damage in patients with cancer, similar to the damage that occurs in autoimmune diseases [28]. Moreover, they can activate dormant cancer cells [29].

Gabriel et al. [30] found that NET formation depends on gasdermin D (GSDMD), a pore-forming protein capable of releasing NE. Moreover, GSDMD, a key mediator of pyroptosis, is an executor of pyroptosis [31]. The classical pathway of pyroptosis is triggered by inflammasome, and the molecular mechanism of inflammasome activation and the induction of pyroptosis are divided into two stages: The first is the initiation stage—the transcription generation of proinflammatory factors, such as NLRP3, caspase-11 and proIL-1β. The second is the activation of the inflammatory complex, which includes members of the NLR protein family, Pro-Caspase-1 and the bridging protein ASC/TMS1. Notably, NLRP3, among the members of the NLR protein family, is the main inflammatory complex in pyroptosis [32]. However, in our previous research, we found six pyroptosis genes associated with the survival of patients, including *NLRP3* [33]. Moreover, recent studies have found that pyroptosis, which is regulated by non-coding RNA and other molecules, affects tumor proliferation, invasion, and metastasis [34]. Therefore, we speculate that the mechanism by which NETs affect the invasion and metastasis of OSCC is linked to pyroptosis and that NLRP3 plays an important role.

Currently, the studies on NETs related to oral cavity have mainly focused on periodontitis and clinical research of OSCC. However, the mechanistic study on the effect of NETs on the occurrence and development of OSCC have not been reported [35–37]. Therefore, we aimed to clarify the potential mechanism of NETs in regulating the tumor invasion and metastasis and further provide evidence of tumor pyroptosis may act as a key contributor for OSCC development.

## Results

### High enrichment of NETs is associated with poor prognosis in OSCC

**Fig. 1:**
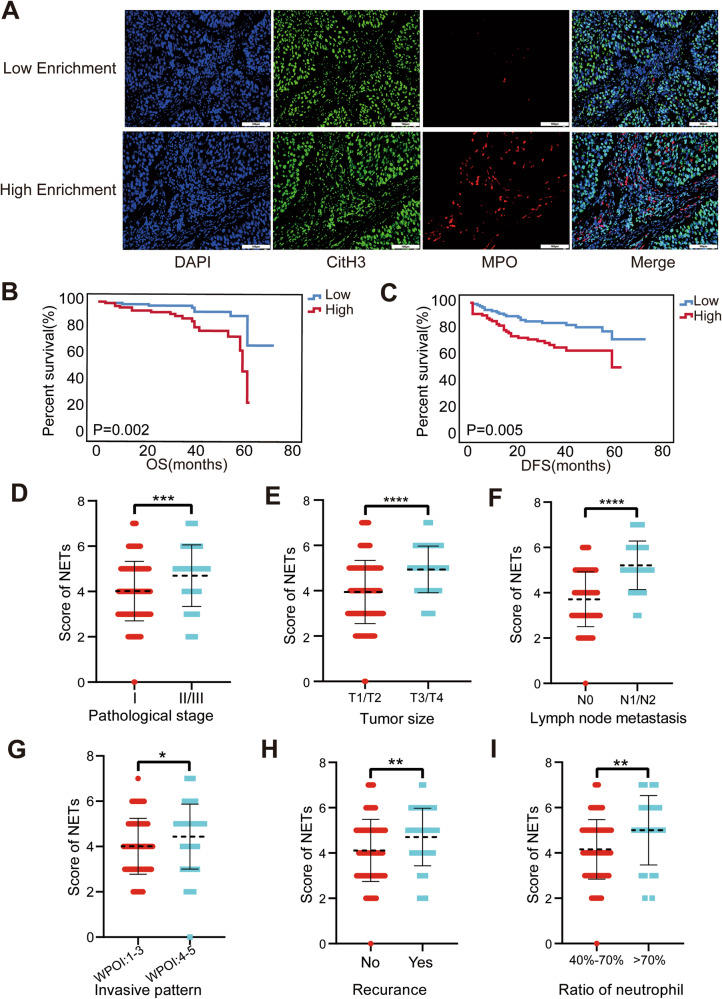
Correlation of NETs with clinical information and prognosis in OSCC patients. **A** Representative multiple immunohistochemical staining pictures about different grades of citH3 and MPO taken under 200× (scale bars = 100 μm). **B** Kaplan–Meier survival analysis about overall survival. **C** Kaplan–Meier survival analysis about disease-free survival. **D**–**I** The correlation analysis between the score of NETs and pathological stage, tumor volume, lymphatic metastasis, infiltration situation, recurrence, and the ratio of neutrophil-lymphocyte. (*P < 0.05, **P < 0.01, ***P < 0.001, ****P < 0.0001). citH3 citrullinated histone H3, NET neutrophil extracellular trap, OSCC oral squamous cell carcinoma, MPO myeloperoxidase.

The activation and recruitment of neutrophils at tumor sites induce the release of web-like structures to form NETs, which are associated with tumor progression in various types of cancers [38, 39]. To explore the potential role of NETs in OSCC development, 205 patients cohort (pathological grade: GI, *n* = 132; GII, *n* = 60; GIII, *n* = 13) were recruited for multiple IHC (mIHC) staining. Given that the formation of NETs can be determined by the co-localization of both citrullinated histone H3 (citH3) and MPO [15], we measured the levels of citH3 and MPO and calculated the overlapping area and intensity of NETs markers in our cohort (Fig. 1A). Subsequently, based on the different enrichment level of NETs, the cohort was divided into high enrichment group (*N* = 94, score ≥ 5) and low enrichment group (*N* = 111, score <5). We found that higher enrichment levels of NETs were associated with worse patient outcomes (Fig. 1B, C), advanced pathological stage, tumor size, lymph node metastasis, worst pattern of invasion, recurrence, and neutrophil-lymphocyte ratio (Fig. 1D–I and Table 1). However, NETs showed no obvious correlation with age, sex, or the number of neutrophils in patients (Fig. S1A–C). These data indicate that prognosis-related NETs are strongly associated with the progression of OSCC.

**Table 1:** Cross analysis of clinical information from OSCC patients and their score of NETs.

Variables	Cases [number, %]	Score of NETs [number, %]		
		Low Group	High Group	*P* value
**Age [years]**				0.818
≤ 60	92 [44.9%]	49 [44.1%]	43 [45.7%]	
> 60	113 [55.1%]	62 [55.9%]	51 [54.3%]	
**Gender**				0.090
Male	122 [59.5%]	72 [64.9%]	50 [53.2%]	
Female	83 [40.5%]	39 [35.1%]	44 [46.8%]	
**Pathological differentiation**				0.001*
I	132 [64.4%]	84 [75.7%]	48 [51.1%]	
II	60 [29.3%]	24 [21.6%]	36 [38.3%]	
III	13 [6.3%]	3 [2.7%]	10 [10.6%]	
**T stage**				<0.001*
T1	36 [17.6%]	27 [24.3%]	9 [9.6%]	
T2	104 [50.7%]	63 [56.8%]	41 [43.6%]	
T3	49 [23.9%]	14 [12.6%]	35 [37.2%]	
T4	16 [7.8%]	7 [6.3%]	9 [9.6%]	
**Lymph node metastasis**				< 0.001*
N0	129 [62.9%]	92 [82.9%]	37 [39.4%]	
N1	41 [20.0%]	7 [6.3%]	34 [36.2%]	
N2	35 [17.1%]	12 [10.8%]	23 [24.5%]	
**Invasion**				0.003*
No	86 [42.0%]	57 [51.4%]	29 [30.9%]	
Yes	119 [58.0%]	54 [48.6%]	65 [69.1%]	
**Recrudescence**				0.014*
No	154 [75.1%]	91 [82.0%]	63 [67.0%]	
Yes	51 [24.9%]	20 [18.0%]	31 [33.0%]	
**Death**				0.010*
No	179 [87.3%]	101 [92.8%]	76 [80.9%]	
Yes	26 [12.7%]	8 [7.2%]	18 [19.1%]	
**Number of neutrophil**				0.461
< 2 × 10^9^	11 [5.4%]	4 [3.6%]	7 [7.4%]	
2 × 10^9^ –7 × 10^9^	188 [91.7%]	104 [93.7%]	84 [89.4%]	
> 7 × 10^9^	6 [2.9%]	3 [2.7%]	3 [3.2%]	
**Neutrophil–lymphocyte ratio**				0.005*
< 50%	19 [9.3%]	11 [9.9%]	8 [8.5%]	
50%–70%	161 [78.5%]	94 [84.7%]	67 [71.3%]	
> 70%	25 [12.2%]	6 [5.4%]	19 [20.2%]	

The *p*-values were obtained using the chi-square test. The asterisks indicate the *p*-values: *< 0.05.

### NETs promote EMT in oral cancer cells

To investigate the effect of NETs in regulating OSCC development, neutrophils from PBMCs of healthy donors were harvested and further treated with phorbol 12-myristate 13-acetate (PMA; 100 nM) to induce NETs formation in vitro. The treated neutrophils were then subjected to an immunofluorescence assay to determine NET formation (Fig. S2A).

To evaluate the potential impact of NETs on modulating the EMT of HNSCC, NETs were collected and utilized as conditioned medium (CM) to stimulate HNSCC cell lines (Cal27, HN6, Fadu, HN4, HN30, HSC3, SCC4, SCC9, and SCC25). Based on the related protein and gene expression changes in HNSCC cells, we noticed that the 100 nM PMA-treated cells exhibited no significant changes in EMT-related markers (Figs. S2B, C, S3, and S4). However, NETs CM promoted EMT in these cancer cells, especially in two tongue squamous cell carcinoma cell lines (HN6 and Cal27) (Figs. S2D–E, S3, and S4). Taken together, these findings indicate that NETs are strong candidates for OSCC development.

### NETs regulate the biological behaviors of OSCC

**Fig. 2:**
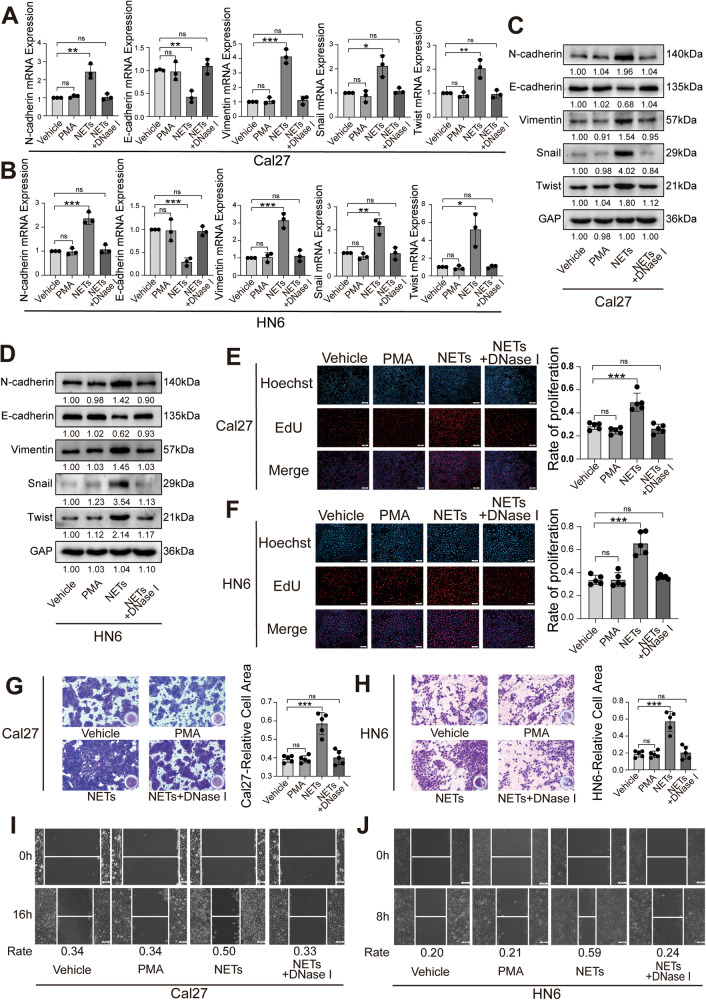
Effect of NETs on the biological behavior of OSCC cells. **A**, **B** The effect of NETs on EMT-related markers in Cal27 and HN6 cells was detected by RT-qPCR. **C**, **D** The effect of NETs on EMT-related markers in Cal27 and HN6 cells was detected by western blot. **E**, **F** The effect of NETs on the proliferation of Cal27 and HN6 cells was detected by EdU proliferation assay. (*n* = 5). (Scale bars=1 mm). **G**, **H** The effect of NETs on the invasion of Cal27 and HN6 cells was detected by Transwell invasion assay (*n* = 5) (scale bars = 200 μm). **I**, **J** The effect of NETs on the migration of Cal27 and HN6 cells was detected by wound healing assay. (scale bars = 100 μm) (ns *P* ≥ 0.05, *P < 0.05, **P < 0.01, ***P < 0.001). NET neutrophil extracellular trap, OSCC oral squamous cell carcinoma.

To further investigate the potential role of NETs in modulating the biological behavior of OSCC, OSCC cells were treated with conditioned media containing NETs. Results of the western blot (WB) and RT-qPCR revealed that NETs regulate EMT-related gene expression at both the mRNA and protein levels, thereby promoting EMT in OSCC. Simultaneously, due to the NET-specific digestive effect of DNase I, no significant modulations of EMT markers in the NETs + DNase I treated group were observed (Fig. 2A–D).

Subsequently, the EdU assay was used to detect the effects of NETs on the regulation of OSCC proliferation. The results showed that proliferation was significantly enhanced in the NET-treated cells (Fig. 2E, F). Moreover, the transwell assays demonstrated that the invasion capacity of OSCC cells was enhanced by NETs (Fig. 2G, H), and the wound healing assay showed that cancer cells treated with NETs exhibited a stronger migratory capacity (Fig. 2I, J). In addition, rescue experiments demonstrated that NET-induced advanced proliferation, invasion, and metastasis of cancer cells were completely abrogated by DNase I. Collectively, these findings indicate that NETs could promote proliferation as well as enhance the migration and invasion behavior of oral cancer cells.

### NETs inhibit the pyroptosis level of OSCC

**Fig. 3:**
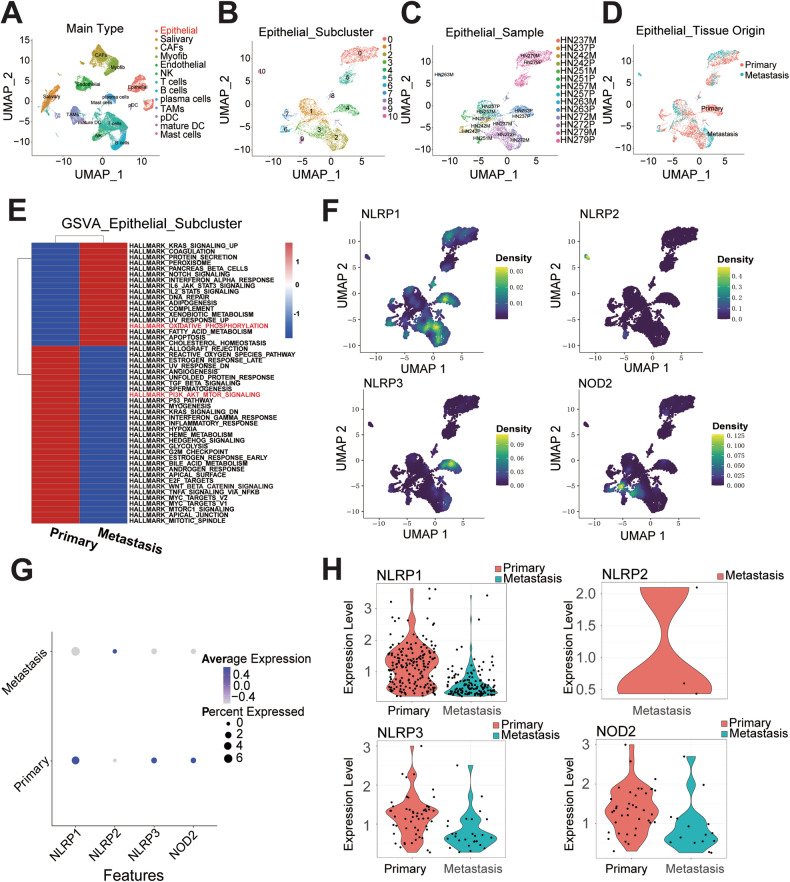
Protective effect of PRGs in preventing OSCC metastasis. **A** Uniform manifold approximation and projection (UMAP) of scRNA-seq with clusters colored and labeled according to inferred cell types from all 53,459 cells separated by primary tumors and metastatic lymph nodes from 7 HNSCC patients. **B**–**D** UMAP of 6115 malignant epithelial cells only, clustered by Seurat clusters (**B**), sample (**C**), and tissue origin (primary/metastatic) (**D**). **E** Heat-map of differentially expressed pathways enrichment between primary malignant epithelial and lymph-node malignant epithelial through Gene Set Variation Analysis (GSVA) algorithm utilizing hallmark genesets. **F**–**H** The density plot (**F**), bubble plot (**G**) and violin plot (**H**) demonstrated the different expression level of PRGs (NLRP1、NLRP2、NLRP3、NOD2) between primary tumor and metastatic tumor. NET neutrophil extracellular trap, OSCC oral squamous cell carcinoma, PRG pyroptosis-related gene.

To better understand the detailed mechanism by which NETs regulate OSCC development, we extracted a subset of cells previously identified as HNSCC epithelial cells from a published scRNA dataset (GSE188737). We regrouped them into dimensionally reduced clusters (Fig. 3A–C), further defined the subgroups by tissue origin (primary cancer cells and metastatic cancer cells) (Fig. 3D), and visualized these subgroups of epithelial cells according to different grouping principles using Uniform Manifold Approximation and Projection (UMAP). We subsequently used the Gene Set Variation Analysis (GSVA) R package to calculate pathway enrichment through the GSVA algorithm, utilizing hallmark gene sets downloaded using the MSIGDBR R package (Fig. 3E). The results demonstrated that the “PI3K-AKT-MTOR” signaling pathway, recently identified as a pyroptosis-related pathway [40, 41], was significantly enriched in primary tumors. However, the “oxidative phosphorylation” signaling was significantly enriched in the metastatic tumors. This result was in line with that of our previous studies, which indicated that mitochondrial function is critical for cancer metastasis [42] and that pyroptosis in cancer cells may serve as a protective factor in preventing metastasis.

Given that the formation relies on gasdermin D (GSDMD), a pore-forming protein capable of puncturing granules to release NE, GSDMD also plays a key role in mediating pyroptosis [30]. However, the membrane pore-forming activity in the gasdermin-N domain of GSDMD is responsible for pyroptosis execution, which performs the necrotic function of pyroptosis [43]. We further hypothesized that pyroptosis may participate in NET-induced modulation of OSCC biological behavior.

Furthermore, based on our previous work, the expression of survival-associated pyroptosis-related genes (PRGs) was shown in the scRNA dataset (Fig. 3F). *NLRP3*, *NOD2*, and *NLRP1* were all highly expressed in primary tumors (Fig. 3G, H), further validating the protective role of PRGs in inhibiting cancer metastasis [44].

**Fig. 4:**
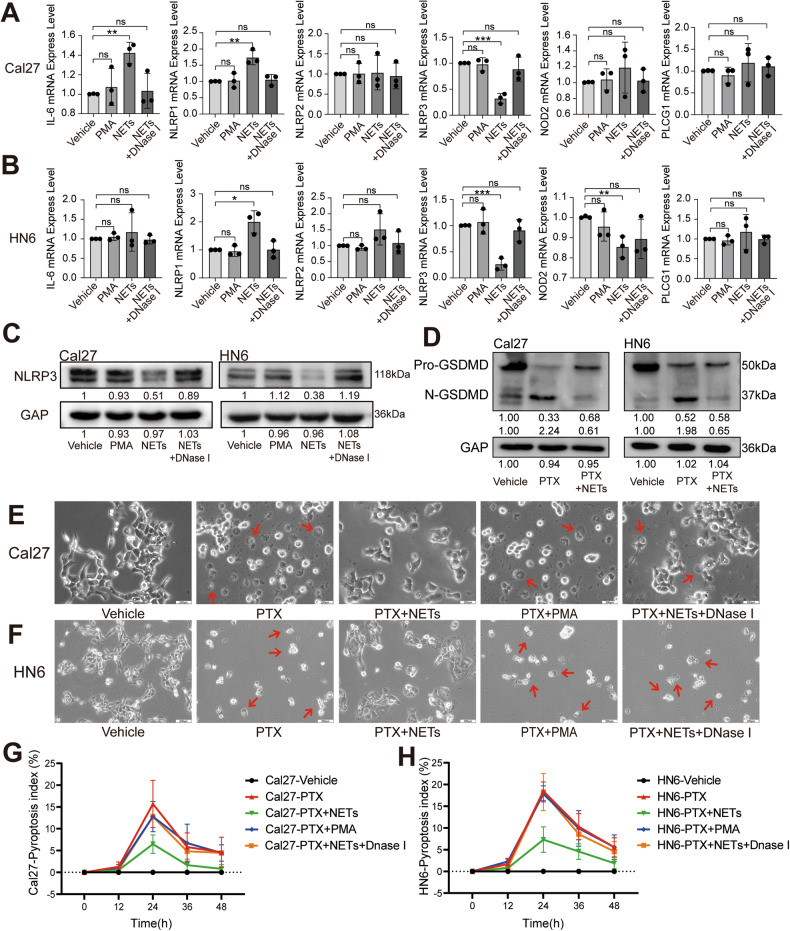
Effect of NETs on the pyroptosis of OSCC. **A**, **B** The effect of NETs on the expression of *IL-6*, *NLRP1*, *NLRP2*, *NLRP3*, *NOD2* and *PLCG1* in Cal27 and HN6 cells was detected by RT-qPCR. **C** The effect of NETs on the expression of NLRP3 in Cal27 and HN6 cells was detected by western blot. **D** The effect of NETs on the gasdermin cleavage level in Cal27 and HN6 cells was detected by western blot. **E**, **F** Representative bright-field microscopy images of Cal27 and HN6 cells after the treatment of NETs; the arrows indicated pyroptotic cells. (Scale bars = 200 μm). **G**, **H** Pyroptosis index of Cal27 and HN6 cells after treatment (*n* = 4). (ns *P* ≥ 0.05, *P < 0.05, **P < 0.01, ***P < 0.001). NET neutrophil extracellular trap, OSCC oral squamous cell carcinoma.

In our previous study, we screened six PRGs, including *IL-6, NLRP1, NLRP2, NLRP3, NOD2*, and *PLCG1*, further demonstrating the importance of PRGs in predicting HNSCC patient outcomes [33]. In the present study, we detected the mRNA expression levels of these genes in the NETs-, PMA-, Nets + DNase I-, and vehicle-treated groups and found that *NLRP3* was downregulated in the NET-treated group (Fig. 4A, B). These results were further validated by WB of Cal27 and HN6 cells (Fig. 4C), indicating that NLRP3 may serve as an important inhibitor of NET-induced OSCC development.

Recent studies have demonstrated that NLRP3 plays a crucial role in regulating cellular pyroptosis [45]. In light of these findings, we speculate that NET-induced downregulation of NLRP3 leads to the inhibition of cellular pyroptosis, thereby promoting OSCC development. According to the results of gasdermin cleavage assays, we found that the pyroptosis was present in OSCC cell lines and the formation of N-GSDMD was reduced under the action of NETs (Fig. 4D). However, based on a previously established pyroptosis evaluation index [46], we found that NETs significantly reduced the cellular pyroptosis levels of Cal27 and HN6 (Fig. 4E–H). Collectively, these data suggested that NETs inhibit pyroptosis in OSCC cells.

### NLRP3-induced the enhancement of pyroptosis inhibits the OSCC development

**Fig. 5:**
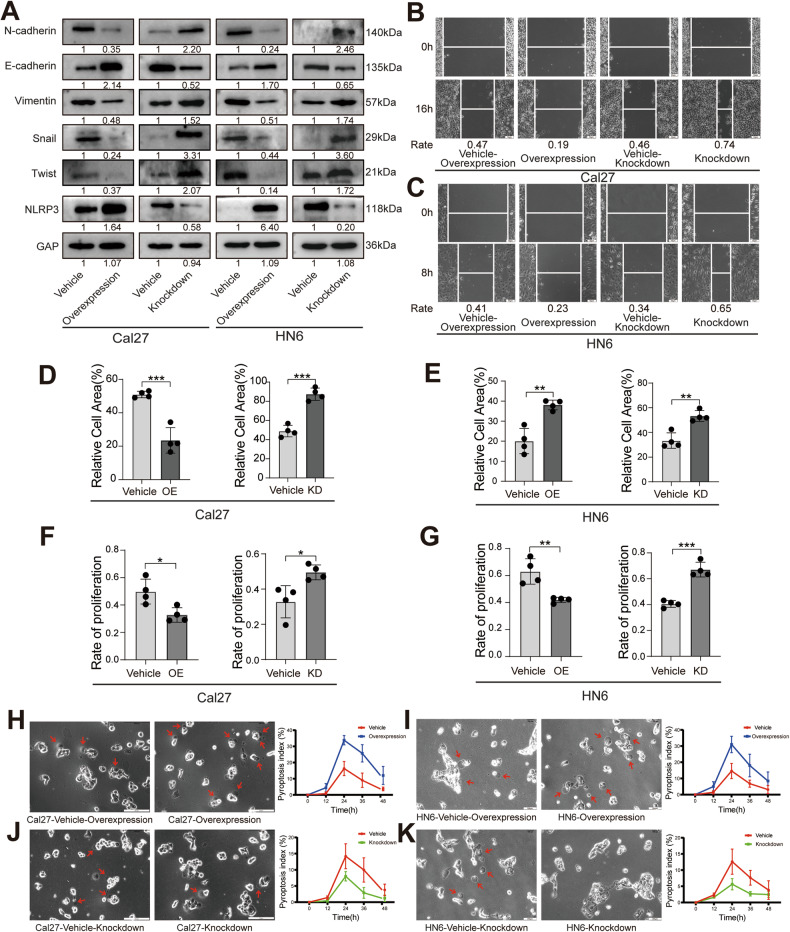
Effects of NLRP3 on the biological behavior of OSCC cells. **A** The transfection efficiency of NLRP3 and the effects of NLRP3 on EMT-related markers in Cal27 and HN6 cells was detected by western blot. **B**, **C** The effects of NLRP3 on the migration of Cal27 and HN6 cells was detected by wound healing assay. (scale bars = 50 μm). **D**, **E** The effect of NLRP3 on the invasion of Cal27 and HN6 cells was detected by Transwell invasion assay. (*n* = 4). **F**, **G** The effect of NLRP3 on the proliferation of Cal27 and HN6 cells was detected by EdU proliferation assay. (*n* = 4). **H**–**K** Representative bright-field microscopy images of Cal27 and HN6 cells after treatment; the arrows indicated pyroptotic cells. And pyroptosis index of Cal27 and HN6 cells after treatment (*n* = 4). (Scale bars = 200 μm). (*P < 0.05, **P < 0.01, ***P < 0.001). NET neutrophil extracellular trap, OSCC oral squamous cell carcinoma.

We further investigated the effect of NLRP3 on the modulation of OSCC biological behavior. We established stable NLRP3 knockdown and overexpression cell lines via lentiviral infection of HN6 and Cal27 cells and found that overexpression of NLRP3 could inhibit the EMT process of OSCC (Fig. 5A). In the invasion and wound healing assays, overexpression of NLRP3 significantly inhibited the invasive and migratory capacities of OSCC (Fig. 5B–E, S5A, B). We used the EdU proliferation assay to further validate that overexpression of NLRP3 inhibited the proliferative capacity of OSCC cells (Fig. 5F, G, S5C, D). Furthermore, we observed that the expression of NLRP3 significantly elevated OSCC pyroptosis (Fig. 5H–K).

**Fig. 6:**
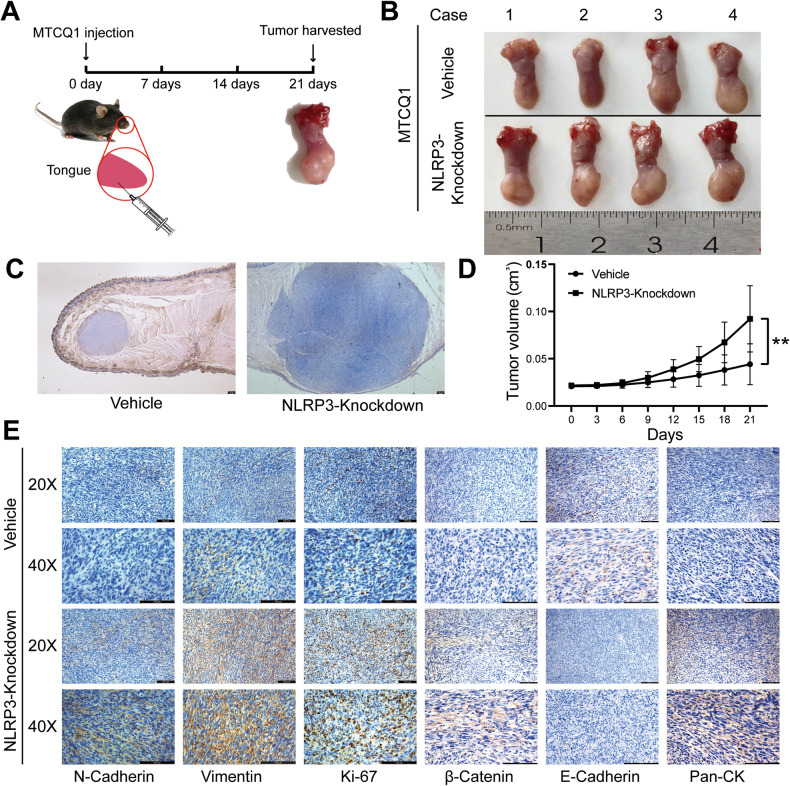
Effect of NLRP3 on OSCC in vivo. **A** Schematic illustration of the experimental design of mouse. (*n* = 4). **B** Tumor tissues were harvested after 21 days. **C** Representative hematoxylin-eosin staining pictures about tumor tissues taken under 25×. **D** Volume of tongues in each group. **E** Representative immunohistochemical staining pictures about E-cadherin, N-cadherin, Vimentin, Ki-67, β-Catenin and pan-Cytokeratin in tumor tissues. (**P < 0.01, scale bars = 100 μm). OSCC Oral squamous cell carcinoma.

Next, we transfected MTCQ1 cells with NLRP3 knockdown constructs. The cells were then injected into the tongues of mice to induce OSCC (Fig. 6A). The results showed that the depletion of NLRP3 significantly promoted tumor growth (Fig. 6A–D). We further used IHC to detect proliferation and EMT markers in tumor tissues and found that the expression of NLRP3 in OSCC exhibited a strong protective effect against OSCC development (Fig. 6E). In conclusion, our results indicate that *NLRP3* serves as an anti-oncogene that induces pyroptosis in OSCC, further inhibiting OSCC development.

### NE in NETs inhibits OSCC pyroptosis through NLRP3

**Fig. 7:**
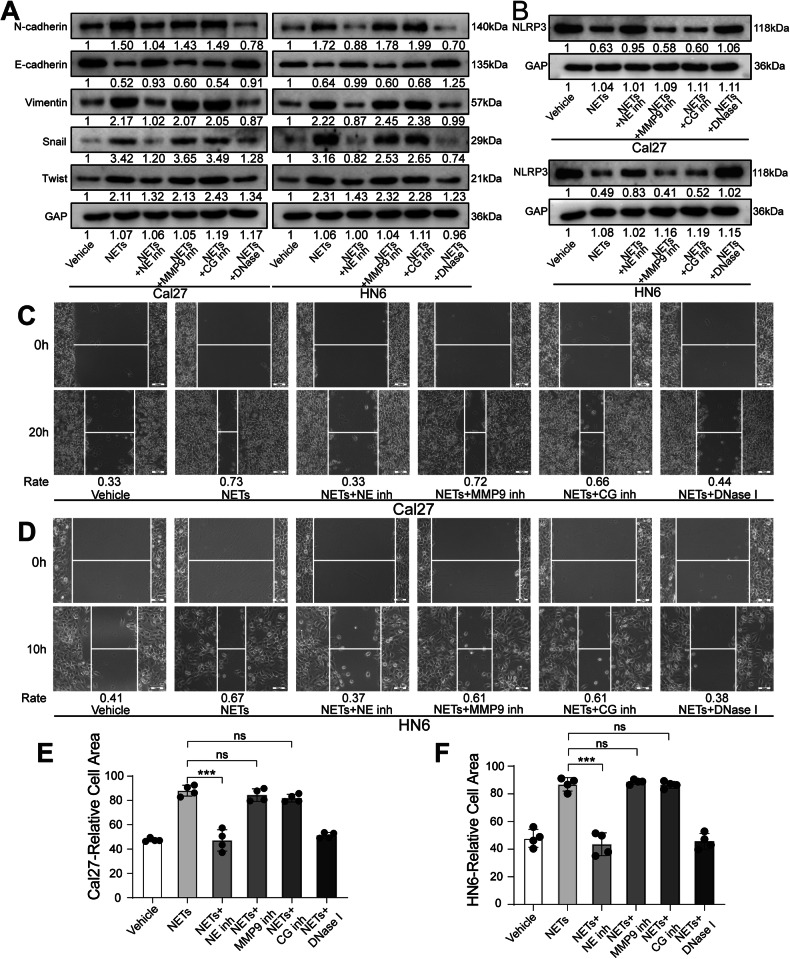
Effect of the main components of NETs on the biological behavior of OSCC cells. **A**, **B** After inhibiting NE, MMP 9 and CG in NETs, western blot was used to detect the EMT-related markers and NLRP3 in Cal27 and HN6 cells. **C**, **D** After inhibiting NE, MMP 9 and CG in NETs, wound healing assay was used to detect the migration of Cal27 and HN6 cells. **E**, **F** After inhibiting NE, MMP 9 and CG in NETs, Transwell invasion assay was used to detect the invasion of Cal27 and HN6 cells. (*n* = 4). (ns *P* ≥ 0.05, ***P < 0.001, scale bars = 100 μm). CG cathepsin G, MMP9 matrix metalloproteinase 9, NE neutrophil elastase, NET neutrophil extracellular trap, OSCC oral squamous cell carcinoma.

Next, to determine the detailed mechanism of NETs in altering OSCC biological behaviors, we tested whether the main components of NETs would play an important role in participating in relevant regulations. NETs have three main secretory granules: neutrophil elastase (NE), matrix metalloproteinase 9 (MMP9), and cathepsin G (CG), which are also associated with decondensed chromatin during NET formation of NETs [15]. We diminished these three components by related inhibitors. Consequently, we noticed that inhibition of NE in NETs could significantly abrogate the EMT process, meanwhile promoting the NLRP3 expression of oral cancer cells (Fig. 7A, B). We also confirmed that the three inhibitor-induced EMT variations in OSCC cells did not occur in the absence of NETs (Fig. S6).

We further confirmed that NE contributes to NET-mediated modulation of OSCC migration and invasion capacity (Fig. 7C–F and S7A, B). Notably, in the EdU proliferation assay, we found that the inhibition of NE, MMP9, and CG had no significant effect on OSCC proliferation (Fig. S7C–F).

By inhibiting the main components of NETs, we confirmed that NE contributed to NET-induced modulation of pyroptosis in OSCC (Figs. S8A–D). Furthermore, we treated stably transfected cell lines (NLRP3-overexpression and knockdown) with PTX, NETs, or an NE inhibitor. We noticed that NE was an indispensable contributor to NETs by regulating NLRP3-mediated inhibition of OSCC pyroptosis (Fig. S8E–H). Collectively, these results suggest that NE acts as the main component of NET-induced inhibition of OSCC pyroptosis in an NLRP3-dependent manner, promoting its invasion and metastasis (Fig. S9).

## Discussion

Neutrophils, the largest number of leukocytes in the circulation, constitute a large proportion of inflammatory cells in the microenvironments of various malignant tumors [47–49]. However, the role of neutrophils in tumors remains controversial. Various studies have suggested that tumor-associated neutrophils (TANs) possess a variety of antitumor properties, including direct cytotoxicity to tumor cells and inhibition of metastases [50–52]. However, many studies have shown that TANs support tumor progression by promoting angiogenesis, stimulating the migration and invasion of tumor cells, and modulating other immune cells [53]. Numerous studies have found that the function of neutrophils in promoting tumor development and progression does not occur through a phagocytic mechanism; nevertheless, it occurs through the formation of NETs in tumors. NETs are net-like structures that trap bacteria, fungi, protozoans and viruses [15]. During their formation, the nuclear membrane of activated neutrophils disintegrates, the plasma membrane disintegrates, nuclear contents are released, chromatin decondenses, and chromosomes containing granule proteins are released into the extracellular space [17]. Zychlinsky et al. [54] speculated that Ewing sarcoma cells could stimulate TAN to form NETs. Richardson et al. [55] found that NETs were substantially increased in patients with colorectal cancer, compared with those in healthy volunteers. In the present study, we found that NETs form in OSCC and are closely associated with clinical data and the prognosis of patients with OSCC (Fig. 1 and S1 and Table 1). Our results indicate that NETs play an important role in OSCC progression. This may provide a new approach for OSCC treatment.

Recently, NETs have been confirmed to be closely associated with tumors [56]. An increasing number of studies have shown that NETs are formed in the tumor microenvironment, participate in the occurrence and development of tumors, and play dual roles in tumor immune editing, tumor progression, tumor metastasis, and tumor-related thrombosis [14, 21]. NETs can exert anti-tumor effects. NET ingredients, such as MPO [22], NE [24, 25], and histones [23], can eliminate tumors and inhibit tumor progression and metastasis [22]. However, NETs exhibit tumor-promoting activities. NET proteases can also degrade the extracellular matrix to promote invasion and metastasis of cancer cells [24, 25]. Furthermore, NETs can trap cancer cells and serve as adhesion substrates, thereby facilitating invasion and metastasis [26, 27]. In this study, we also found that NETs could promote EMT in oral cancer cells (Figs. S2–S4) and regulate the biological behavior of OSCC (Fig. 2). Thus, these results confirm that NETs exert tumor-promoting effects in OSCC. However, further studies are required to elucidate the specific mechanism.

Pyroptosis, a mode of programmed cell death, has been found to have an important impact on tumor growth and inhibition, with a thorough study of its mechanism of occurrence [57]. According to previous reports, the classical pyroptosis pathway is triggered by the inflammasome, and NLRP3 is the major inflammatory complex involved in the mechanism [32]. In gastric cancer, LncRNA *ADAMTS9-AS2* activates NLRP3-mediated pyroptosis by adsorbing miR-223-3p, thereby serving as a tumor suppressor and enhancing cisplatin sensitivity [58]. Dysregulated expression of the NLRP3 inflammasome has been implicated in tumor progression in hepatocellular carcinoma [59]. Our previous study identified six pyroptosis-related genes associated with the survival of patients, including *NLRP3* [33]. The relationship between NLRP3 expression and OSCC was also investigated. We identified for the first time that NLRP3-induced enhancement of pyroptosis inhibited OSCC development (Figs. 3–6 and S5). NE, which is the main component of NETs, has an important impact on NET function [15]. The expression and activity are increased in many types of tumors. In the A549 cell line, NE could enhance the proliferation capacity [60]. NE affects the adhesion ability of tumor cells in pancreatic ductal adenocarcinoma. In this study, we confirmed that NE serves as the main component of NET-induced inhibition of OSCC pyroptosis in an NLRP3-dependent manner and regulates the invasion and metastasis of OSCC (Fig. 7 and S6–S8). Based on these results, we elucidated the specific mechanisms by which NETs promote OSCC invasion and metastasis.

However, Limitations of the present study is that specific mechanisms by which NE affects NLRP3-induced pyroptosis were not identified and animal experiments only investigated the effects of NLRP3 in OSCC. Therefore, further in-depth mechanistic and animal investigations are necessary. Moreover, whether drugs that inhibit or degrade NETs, such as inhibitors of NE and PAD 4 or DNase I, can play an important role in tumor development and progression needs to be explored further.

This study is the first to clarify the specific mechanism by which NETs promote the invasion and metastasis of OSCC and to link NETs to pyroptosis in tumors, demonstrating that NE in NETs can promote the invasion and metastasis of OSCC by inhibiting NLRP3-induced pyroptosis. Our findings can aid in providing a new therapeutic approach for the treatment of OSCC.

## Methods

### Patients and samples

Overall, 205 paraffin-embedded tissue samples from patients with OSCC were obtained from the Affiliated Stomatological Hospital of Nanjing Medical University (Nanjing, China). All patients had clear pathological diagnoses, complete clinical data, and well-preserved pathological tissues. None of the patients received radiotherapy or chemotherapy before surgery. The pathological stages of the patients were classified according to the WHO categories, and the clinical stage and TNM classification were classified according to the Union for International Cancer Control. The experimental content and protocol of this study were reviewed and approved by the Stomatology Ethics Committee of Nanjing Medical University (No. (1029)848), and the informed consent has been obtained from all patients.

### Multiple immunohistochemical staining

mIHC was performed using the PANO 4-plex kit (Panovue, Beijing, China). Briefly, after dewaxing, hydration, antigen repair (citrate sodium, Solarbio, Beijing, China), nonspecific antibody blocking, and incubation with primary and secondary antibodies, PPD520, PPD570, and tyramide signal amplification reagents were used to amplify the stained signals. The following primary antibodies were used for mIHC staining: Anti-Histone H3 (1:30000; Novus Biologicals, Abingdon, United Kingdom) and anti-myeloperoxidase (MPO; 1:1000, Abcam, Cambridge, United Kingdom).

Semi-quantitative analysis was performed according to IHC scores [61, 62]. After a comprehensive evaluation of the overlapping area of MPO and citH3, the proportion of stained cells per field was scored as 0, 1, 2, or 3 if positive staining was not observed, < 10% positive staining, 10–30% positive staining, or > 30% positive staining, respectively. IHC scoring of overlapping intensities was classified into four categories (0, 1, 2, and 3) according to the staining intensity (none, weak, moderate, and strong). Finally, the scores were summed to obtain the overall score. The enrichment level of NETs was evaluated by IHC scores, and the cohort was divided into high enrichment group (*N* = 94, score ≥ 5) and low enrichment group (*N* = 111, score < 5) according to the IHC scores.

### Extraction of neutrophils and induction of NETs formation

Peripheral blood samples were collected from healthy adult volunteers in EDTA anticoagulation tubes. Neutrophils were isolated using the human neutrophil isosolution KIT (Tbdscience, Tianjin, China), according to the instructions of the manufacturer. Isolated neutrophils were cultured in RPMI 1640 medium (Invitrogen, Carlsbad, CA, USA) containing 0.2% BSA (Sigma-Aldrich, St. Louis, MO, USA). Methylene blue staining was used to verify sample purity. 95% of cells were granulocytes with multi-lobar nuclei. Trypan blue staining was used to verify >95% cell viability and determine the final neutrophil yield.

To induce NET formation, the isolated neutrophils were seeded in 12-well plates (2 × 10^5^ cells/well) for 4 h. Where indicated, PMA (100 nM; MedChemExpress, NJ, USA) was added to media [63]. NETs were isolated as previously described [64]. Briefly, cold PBS was used to wash the bottom of each dish to collect all adherent material from the bottom. After centrifuging for 10 min at 450 × *g* at 4 °C, neutrophils and any remaining cells pelleted at the bottom, leaving a cell-free NET-rich supernatant. Then, the supernatant was spun for 10 min at 18,000 × *g* at 4 °C. This allowed all DNA to pellet. Finally, these pellets were resuspended in sterile DMEM, and quantified using NanoDrop Lite Spectrophotometer (Thermo Fisher Scientific, Waltham, MA, USA). Isolated NETs were kept at 4 °C for no more than 24 h.

### Cellular immunofluorescence assay

To assess NETs formation, neutrophils (2 × 10^4^ cells) were seeded on coverslips coated with poly-L-lysine (Sigma-Aldrich) in 24-well plates for 4 h after adding PMA (100 nM) or PMA (100 nM) + Deoxyribonuclease I (DNase I; 1 µg/mL; MedChemExpress) at 37 °C. Neutrophils were then fixed with a 4% paraformaldehyde (PFA, Biosharp, Guangzhou, China) solution for 15 min at room temperature (RT), washed twice with PBS, and permeabilized in 0.5% Triton X-100 (Beyotime, Shanghai, China) for 20 min. Cells were blocked with goat serum (Boster Biological Technology, Wuhan, China) for 1.5 h at 37 °C, and incubated with primary antibody against myeloperoxidase (MPO; 1:100; GeneTex, Irvine, CA, USA) and histone H3 (1 µg/mL; Abcam) overnight at 4 °C. After three washes in PBS, cells were incubated with fluorochrome-conjugated secondary antibodies (Proteintech, Chicago, IL, USA) for 1 h at 37 °C, and counterstained with 4’,6-diamidino-2-phenylindole (DAPI, Beyotime) before mounting. Cells were observed and photographed using a fluorescence microscope (Leica Microsystems, Germany).

### Cells culture and cell lines

Cal27, HN6, HN4, HN30, SCC4, SCC9, SCC25, Fadu, and HSC3 human head and neck squamous cell carcinoma cells were purchased from the American Type Culture Collection (USA), and the mouse 4NQO-induced tongue cancer cell line MTCQ1 (BCRC 50620) was obtained from the Bioresource Collection and Research Center. Cal27, HN6, HN4, HN30, FaDu, HSC3, and MTCQ1 cell lines were maintained in DMEM (Invitrogen) containing 10% FBS (ScienCell, San Diego, CA, USA) and 1% penicillin/streptomycin (NCM Biotech, Suzhou, China) at 37 °C in a 5% CO_2_-humidified incubator. The SCC4, SCC9, and SCC25 were maintained in F12 (Invitrogen) containing 10% FBS (ScienCell), 1% penicillin/streptomycin (NCM biotech, China), 0.5% sodium pyruvate (Sigma-Aldrich) and 400 ng/mL Hydrocortisone (Sigma-Aldrich) at 37 °C in a 5% CO_2_-humidified incubator. Moreover, the three inhibitors used to cell lines were NE inhibitor (80 µM, Abcam), CG inhibitor (10 µM, Abcam), and MMP9 inhibitor (25 µM, Abcam).

### Western blot

WB was performed according to standard procedures (BIO-RAD, Hercules, CA, USA). Briefly, cells with or without treatment were harvested and lysed with RIPA buffer (Beyotime) containing the proteinase inhibitor PMSF) (1 mM, Thermo Fisher Scientific). Protein concentration was determined using a BCA Protein Assay Kit (Beyotime). Equal amounts of protein were separated by SDS-PAGE (10%) and transferred to PVDF membranes (Millipore, Billerica, MA, USA). The membranes were blocked with 5% skim milk at RT for 2 h and incubated at 4 °C overnight with primary antibodies against GAPDH (1:8000; Proteintech) as a control, followed by horseradish peroxidase-conjugated secondary antibodies (Proteintech) for 1 h at RT. Primary antibodies including E-cadherin (1:1000; Cell Signaling Technology, CST, Boston, MA, USA), N-cadherin (1:1000; CST), Vimentin (1:1000; CST), Snail (1:1000; CST), TWIST (1:1000; CST), NLRP3 (1:1000; CST), ZEB1 (1:1000; CST), β-Catenin (1:1000; CST) and SMAD2/3 (1:1000; CST). Specific bands were detected using ECL chemiluminescence reagents (Millipore) and visualized using a Fusion Edge Multifunctional Imaging System (Vilber, France). Analyses of the bands were performed using ImageJ software (V1.8.0.112).

### RNA isolation and quantitative reverse transcription-quantitative PCR (RT-qPCR)

Total RNA was extracted from the cells using the TRIzol reagent (Invitrogen), and equal quantities of cDNA were reverse transcribed using Superscript (Vazyme, Nanjing, China) according to the manufacturer’s instructions. qRT-PCR was performed using a PCR System QuantStudio7 (Applied Biosystems, USA) with SYBR Green Master Mix (Vazyme). The primers used are listed in Supplementary Table 1.

### EdU Cell Proliferation Detection

Cell proliferation assays were performed using the Cell-Light^TM^ EdU Apollo In Vitro Kit (RiboBio, Guangzhou, China). Cancer cells (1 × 10^5^) were seeded in 96-well plates and treated according to the experimental requirements. Subsequently, EdU medium was added and the cells were incubated for 2 h. After washing the cells twice with PBS, they were fixed with 4% PFA solution (Biosharp) for 30 min. Cells were then incubated with glycine (2 mg/ml; Biofroxx, Germany) for 5 min and permeabilized with 0.5% Triton X-100 (Beyotime) for 10 min. After adding 1 × Apollo^®^ staining solution (RiboBio) and incubating for 30 min at light avoidance and RT, the cells were washed three times with 0.5% TritonX-100. Finally, a 1 × Hoechst 33342 reaction solution (RiboBio) was added and incubated for 30 min with light avoidance at RT. The cells were imaged using a microscope (Leica Microsystems) and counted in four randomly selected fields.

### Cell invasion and wound healing assays

Cell invasion assays were performed using the Boyden chamber invasion method. A Boyden chamber (Millipore) containing 8-μm pores was coated with matrigel (Corning, Corning, NY, USA). Cancer cells (1 × 10^5^) were seeded in the upper chamber with or without the indicated treatments, and complete medium containing 10% FBS was added to the lower chamber. After incubation in 37 °C for 24 h under 5% CO_2_ and 95% air, the non-invading tumor cells were wiped off the upper surface of the membrane, and the cells on the bottom side of the membrane were fixed in 4% PFA for 20 min, then stained with crystal violet (Beyotime). Cells attached to the lower layer were imaged using a microscope (Olympus, Tokyo, Japan) and counted in five randomly selected fields.

For wound healing assays, tumor cells were grown to 80–90% confluence in 6-well tissue culture plates. Cell monolayers were manually wounded by scraping cells with a 10 μL pipette tip. The debris was removed, and the edge of the scratch was smoothed by washing the cells once with medium. Cells were then cultured in culture medium without FBS to minimize the effect of cell proliferation on migration and treated as indicated. The wound width was measured at different time intervals to evaluate the wound-healing ability of OSCC cells.

### Single-cell RNA sequencing data processing

#### Data acquisition

Publicly available HNSCC single-cell RNA sequencing (scRNA-seq) datasets were retrieved from the GSE188737 dataset. Tumor cells used for scRNA-seq were derived from patients with locally advanced HPV-negative HNSCCS of the primary and cervical lymph nodes. The scRNA-seq data were preprocessed, including quality control, normalization, dimensionality reduction, and major cell type annotation.

#### Sub-clustering of epithelial cells

From a complete dataset, we isolated subsets of cells that were identified as epithelial cells based on broad clustering. In addition, the inferred copy number variation analysis of this epithelial cell population showed that 95% of the cells had significant copy number changes, confirming that this subpopulation was mainly composed of cancer cells [65]. Highly variable genes were identified using an appropriate threshold for mean expression and dispersion (variance/mean). Principal component analysis was performed on approximately 3000 variable genes. To perform clustering and annotate each cluster by tissue origin, we used the function FindClusters on PCs = 1:20 with a resolution of 0.5. For visualization, the dimensionality of the dataset was further reduced using a UMAP with the Seurat function RunUMAP. The UMAP visualizations were then grouped by tissue origin. We used the GSVA R package to calculate pathway enrichment through Gene Set Variation Analysis (GSVA) algorithm, utilizing hallmark genesets downloaded by the MSIGDBR R package. In addition, FetchData was used to search for differences in the expression of pyrogenic genes between the primary and metastatic groups, and the results are shown as density, violin, and bubble graphs.

### In vitro pyroptosis index evaluation

Cal27 and HN6 cells were seeded in 6-well plates (5 × 10^4^ cells/well) and incubated for 12 h. PTX (Paclitaxel, 30 g/mL, MCE) was added to each well for 48 h. The cell morphology was observed using a microscope (Leica Microsystems) at 12, 24, 36, and 48 h after treatment, and balloon-like cells were considered pyroptotic cells. The cells were imaged using a microscope (Leica Microsystems) and counted in four randomly selected fields. The ratio of the pyroptotic cell number to the total cell number in a selected field multiplied by 100 was used as the pyroptosis index for each treatment at the indicated time points [46].

### Lentiviral construct and transfection

NLRP3 lentiviral vectors were constructed by GeneChem Co. Ltd. (Shanghai, China). OSCC cells were transfected with a specific shRNA lentivirus targeting NLRP3 knockdown, and the scrambled sequence was used as a negative control. The same procedure was used to overexpression NLRP3. All transfection procedures were performed according to manufacturer’s instructions. Cells in the negative control (NC) group (OSCC-NC) and the knock-down (KD) or over-express (OE) groups (OSCC-KD and OSCC-OE) were cultured at 37 °C in a 5% CO_2_ incubator and transferred to complete medium 12–16 h after transfection. Stable cells were selected using 2 μg/mL puromycin. Fluorescence microscopy was used to observe the transfection efficiency, and western blotting was used to estimate the efficiency of NLRP3 knockdown or overexpression.

### Mouse experiments

C57BL/6 mice aged 6–7 weeks were used to establish an OSCC mouse model. Stably transfected MTCQ1 cells were centrifuged and resuspended in RPMI-1640 medium and injected with 1 × 10^6^ cancer cells into the tongues of the mice. Mouse tongue volume was measured every 3 days. Three weeks after injection, the tongues were harvested for specimen fixation and observation. Subsequently, relevant indicators were detected using IHC. The primary antibodies used included E-cadherin (1:1000; CST), N-cadherin (1:1000; CST), vimentin (1:1000; CST), β-catenin (1:1000; CST), Ki-67 (1:1000; CST), and pan-cytokeratin (1:100; Santa Cruz Biotechnology, USA).

### Statistics

All statistical analyses were performed using IBM SPSS statistics for windows (version 26.0). Student’s *t*-test and Mann–Whitney U-test were used to evaluate differences in variables between the two groups. Pearson’s correlation analysis was performed to evaluate the correlation between NETs scores and clinical and prognostic information. The results of the survival analysis, which were based on Kaplan–Meier analysis, were compared using the log-rank test. Statistical significance was set at *P* < 0.05.

### Supplementary information

Supplementary Materials

original western blots

IHC scoring data

## Data Availability

All data generated or analyzed during this study are included in this published article and its supplementary information files.
