# Supplementary material for: Neutrophil extracellular traps promote invasion and metastasis via NLRP3-mediated oral squamous cell carcinoma pyroptosis inhibition
Source: Cell Death Discov. 2024 May 2;10:214. doi: 10.1038/s41420-024-01982-9 (PMC11066066; doi:10.1038/s41420-024-01982-9)
Supplement: Supplementary file 1 — Supplementary Materials [file 41420_2024_1982_MOESM1_ESM.docx]

**Supplementary Table**

**Table S1: Primer sequences for qRT-PCR**

| Name | F (5’-3’) | R (5’-3’) |
| --- | --- | --- |
| GAPDH | GAAGGTGAAGGTCGGAGTC | GAGATGGTGATGGGATTTC |
| E-cadherin | TACACTGCCCAGGAGCCAGA | TGGCACCAGTGTCCGGATTA |
| N-cadherin | GACAATGCCCCTCAAGTGTT | CCATTAAGCCGAGTGATGGT |
| Vimentin | TGAGTACCGGAGACAGGTGCAG | TAGCAGCTTCAACGGCAAAGTTC |
| SNAIL | GACCACTATGCCGCGCTCTT | TCGCTGTAGTTAGGCTTCCGATT |
| Twist | CGGGAGTCCGCAGTCTTA | CCCATGTGATTCGATGCGT |
| ZEB1 | TACCAGAGGATGACCTGCCA | TGCCCTTCCTTTCCTGTGTC |
| β-Catenin | AAGTTCTTGGCTATTACGACA | GTCACGAGGTACGACCTCAGAT |
| NLRP1 | AGCTTCTGCTCGCCAATAAAG | CCAGGTATGGAGGGCTAGGT |
| NLRP2 | TTCTGCGTCAAGCACTGTCG | GGATCTCTCAACCTCGGCGT |
| NLRP3 | AGCACTAATCAGAATCTCACGCA | TGTCTAATTCCAACACCTGAAGC |
| PLCG1 | CTCTATGGAATGGAATTTCGCC | GGAGCCACCTCTCAATCTGC |
| NOD2 | CAATGACGATGCGGACACTG | GCTGAATGGGAAGACAAAGAGAA |
| IL-6 | TGCCAGCCTGCTGACGAA | AGCTGCGCAGAATGAGATGA |

**Supplementary Figures**

**
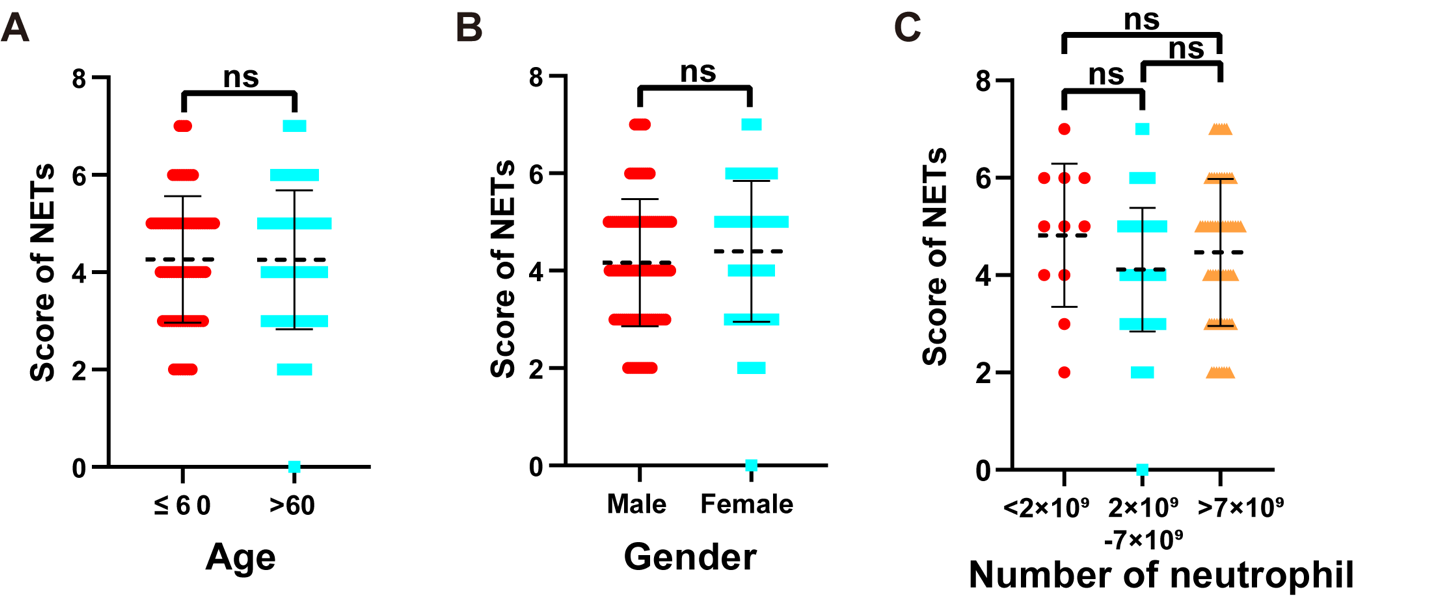
**

**Figure S1. Correlation of NETs with clinical information in OSCC patients.** (A-C). The correlation analysis between the score of NETs and age, gender and number of neutrophils. (ns P≥0.05).

**
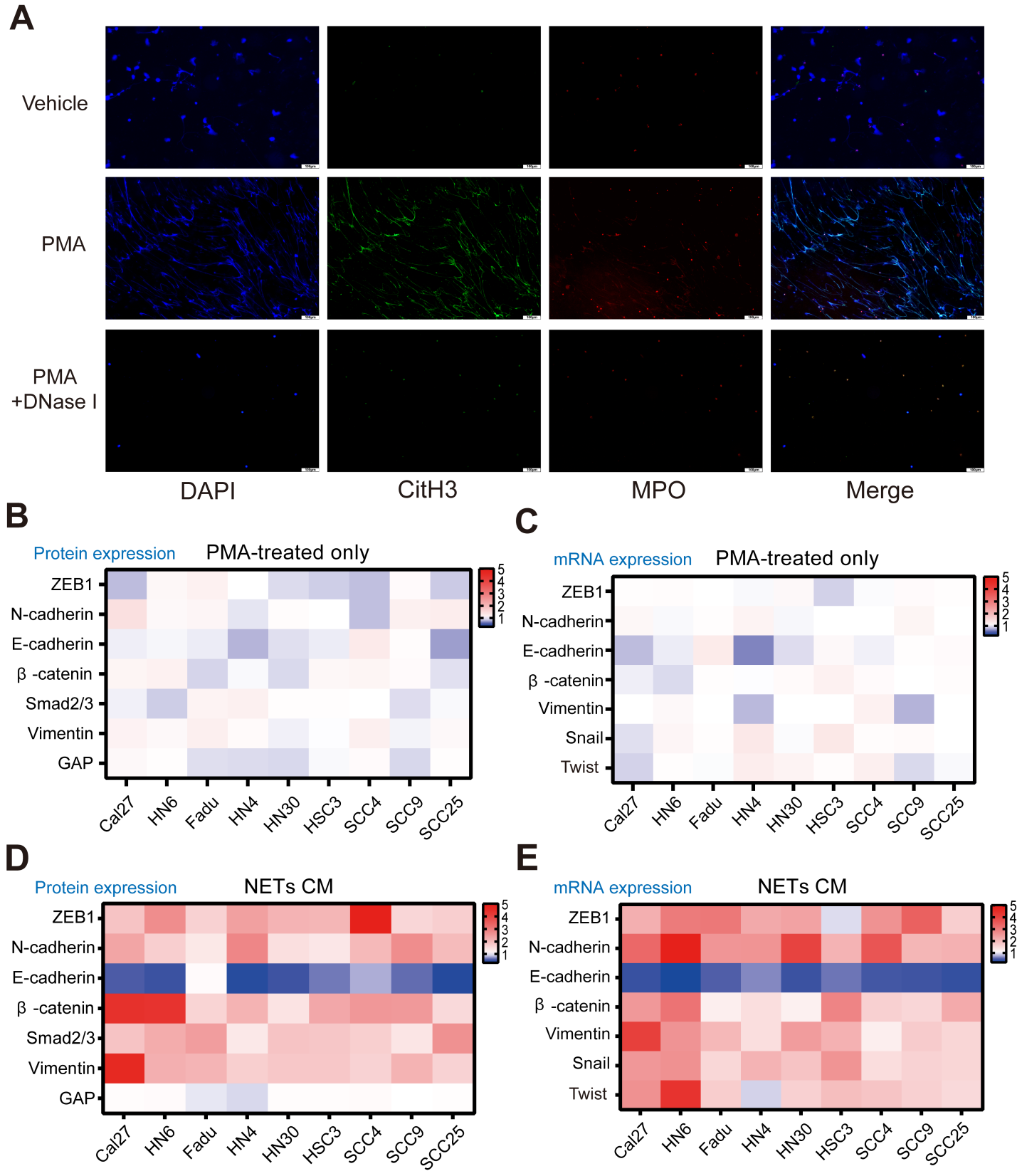
**

**Figure S2. NETs can be induced by 100nM PMA.** (A). Representative immunofluorescent staining to determine the formation of NETs, the formation of NETs can be dismantled by DNase Ⅰ, due to the NET-DNA scaffolds of NETs can be digested by deoxyribonucleases. (scale bars=100 μm). (B–E). Heat-map from Figure S3 and Figure S4. NET, neutrophil extracellular trap; PMA, phorbol 12-myristate 13-acetate.

**
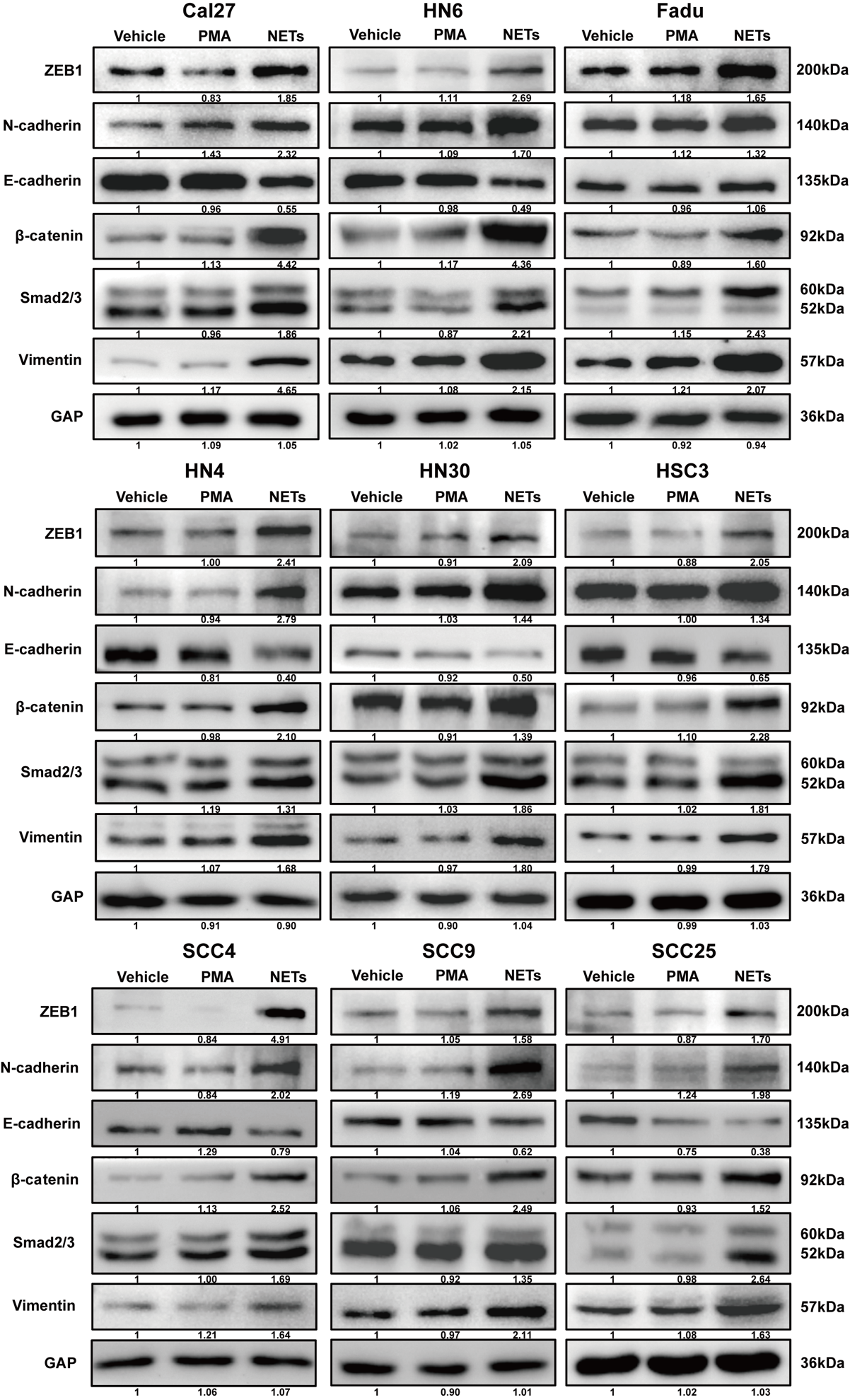
**

**Figure S3. The effect of NETs on EMT-related markers in nine cell lines was detected by western blot.**

**
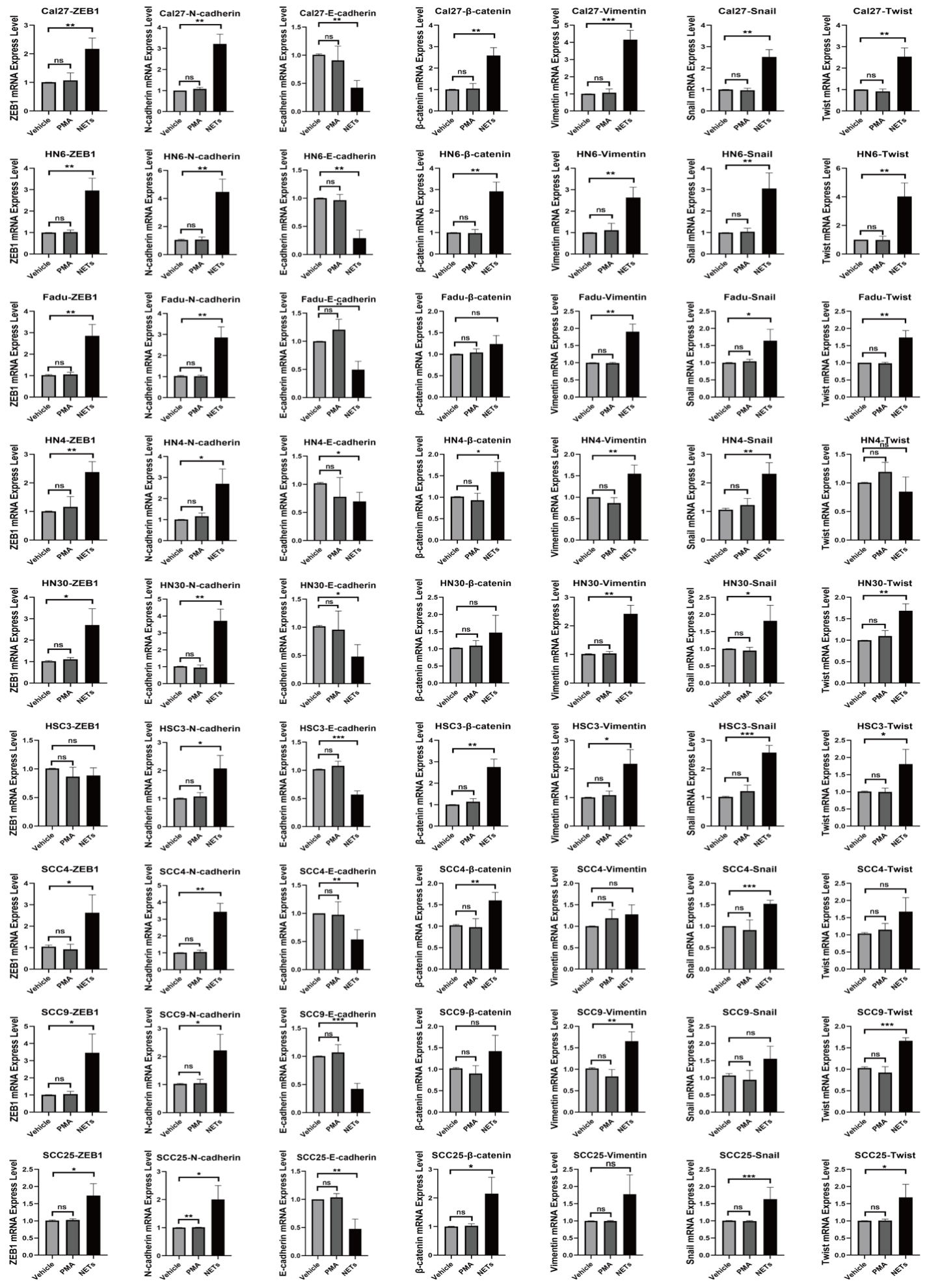
**

**Figure S4. The effect of NETs on EMT-related markers in nine cell lines was detected by RT-qPCR.**

**
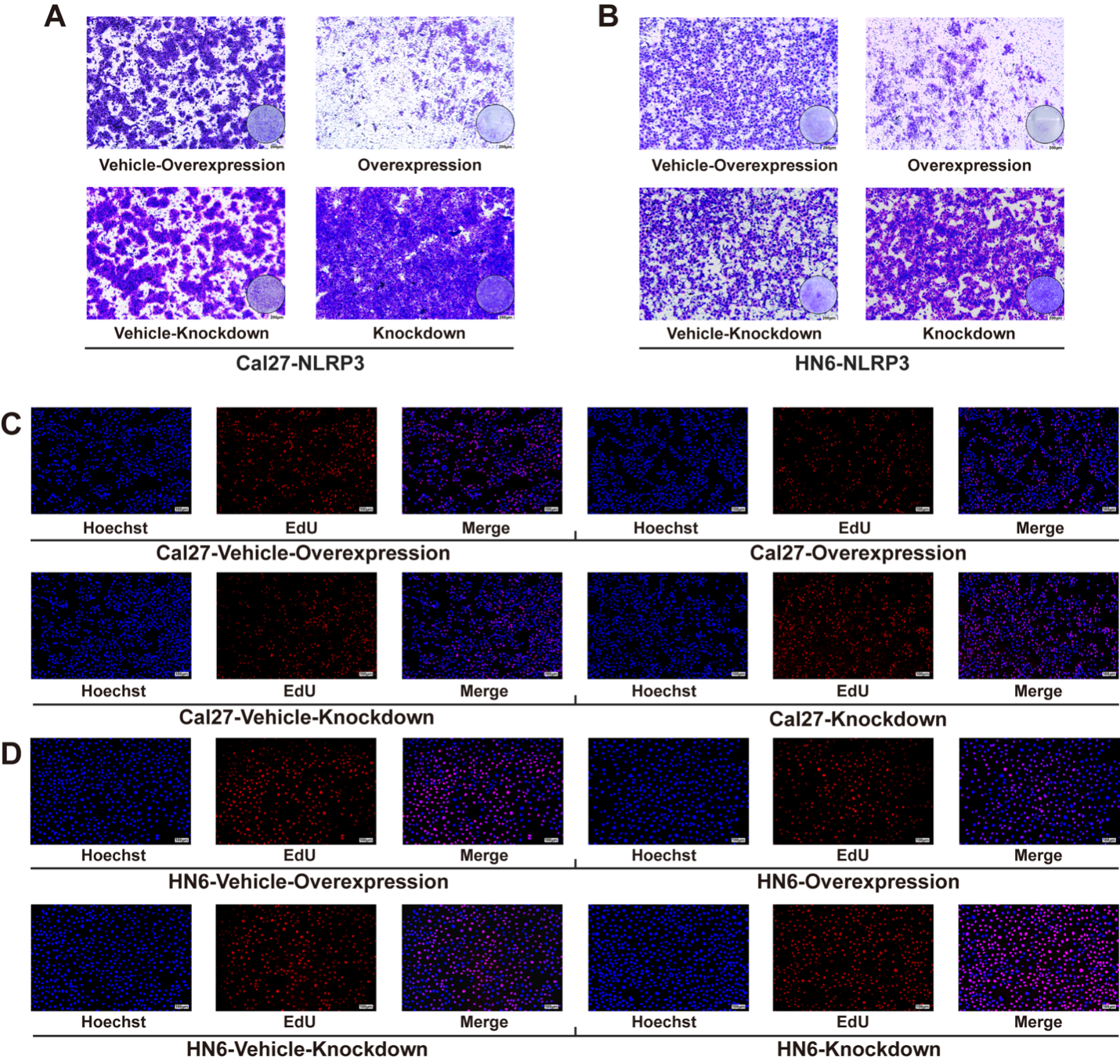
**

**Figure S5. The effect of NLRP3 on the biological behavior of OSCC cells.** (A-B). The effect of NLRP3 on the invasion of Cal27 and HN6 cells was detected by Transwell invasion assay. (scale bars=200 μm). . (C-D). The effect of NLRP3 on the proliferation of Cal27 and HN6 cells was detected by EdU proliferation assay. (scale bars=100 μm). .

**
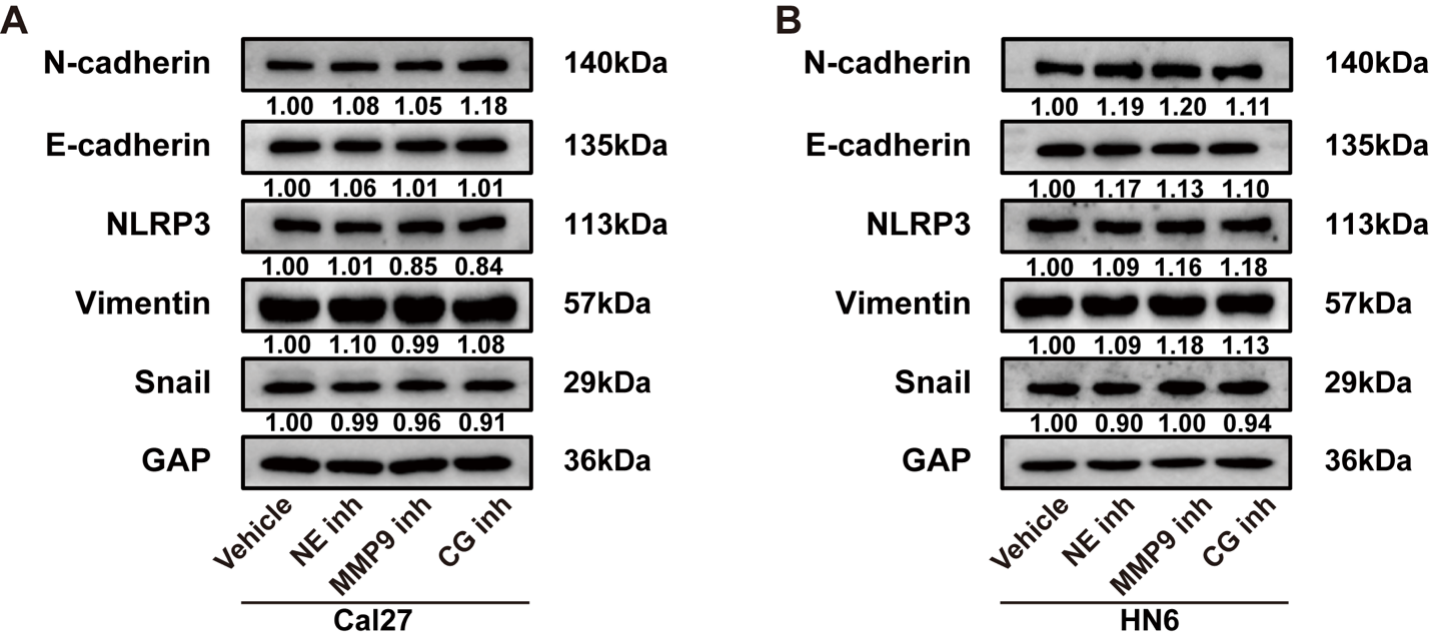
**

**FigureS6. The effect of inhibitors on the OSCC.** (A-B). After Cal27 and HN6 cells were treated with the inhibitors of the NE, MMP9 and CG, the EMT-related markers was detected by western blot.

**
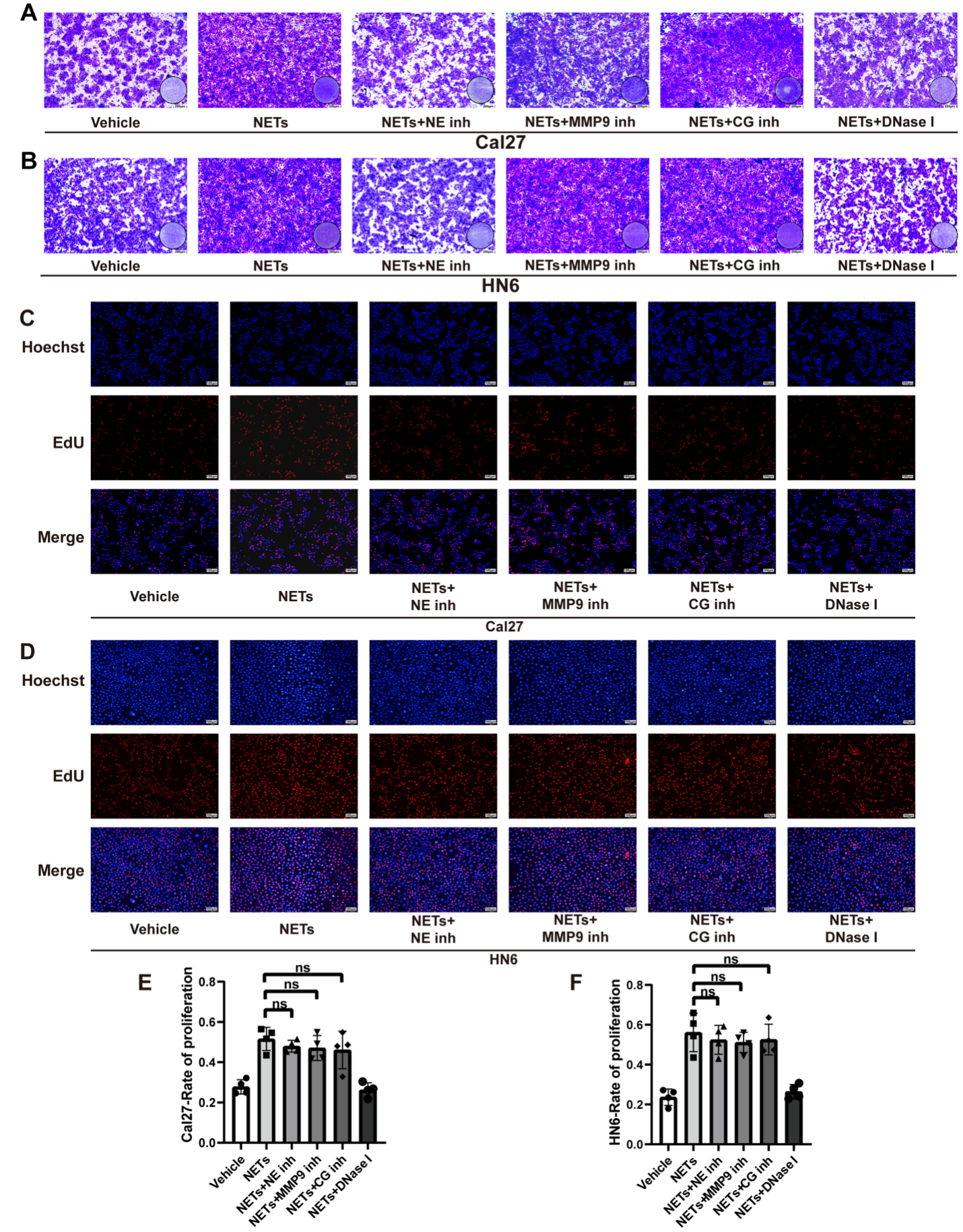
**

**Figure S7. The effect of the main components of NETs on the biological behavior of OSCC cells.** (A-B). After inhibiting NE, MMP 9 and CG in NETs, Transwell invasion assay was used to detecte the invasion of Cal27 and HN6 cells. (Scale bars=200 μm). (C-F). After inhibiting NE, MMP 9 and CG in NETs, EdU proliferation assay was used to detecte the proliferation of Cal27 and HN6 cells. (n=4). (Scale bars=100 μm). (ns P≥0.05).


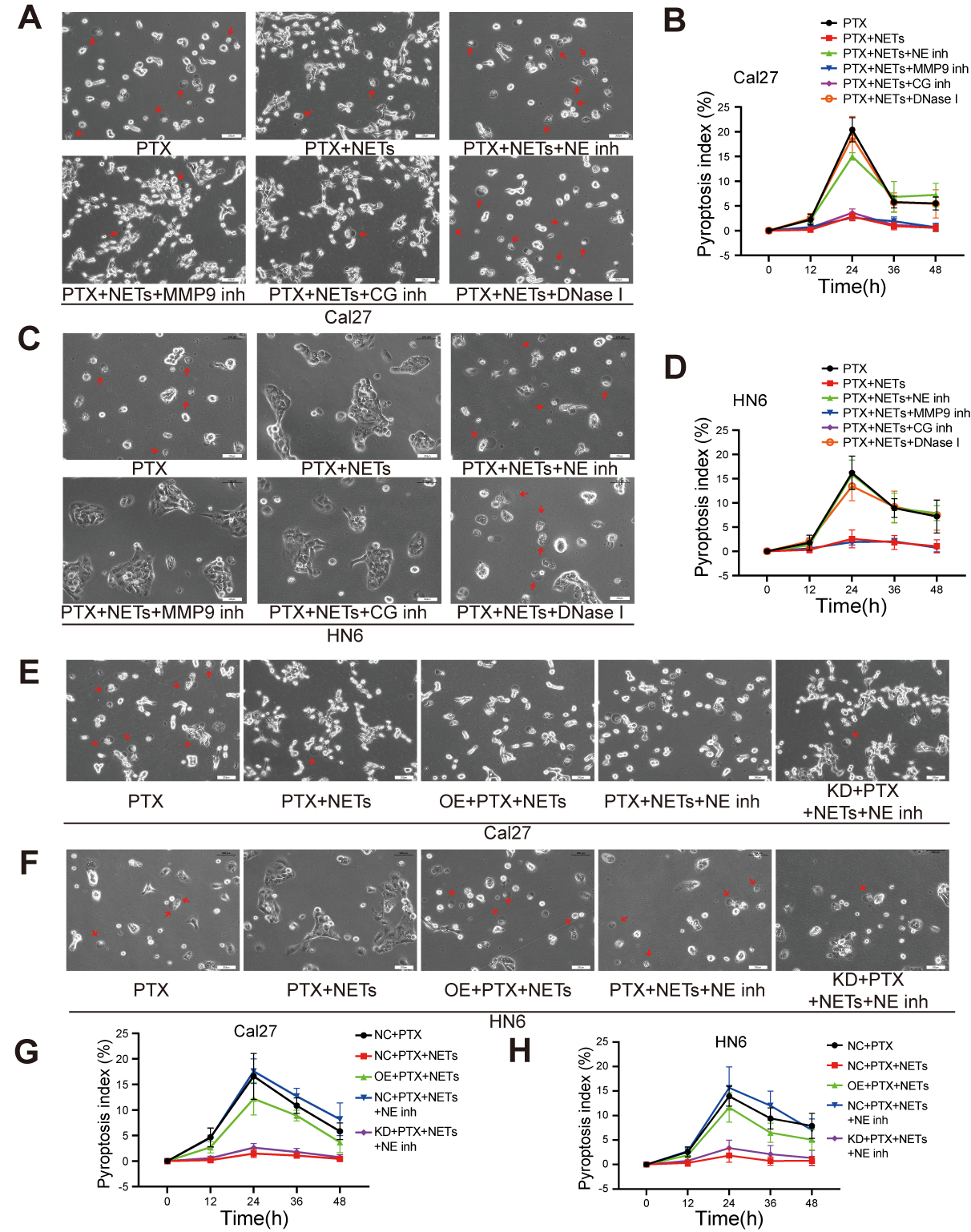


**Figure S8. Effect of the main components of NETs on the pyroptosis of OSCC.** (A–D). After inhibiting NE, MMP 9 and CG in NETs, representative bright-field microscopy images and pyroptosis index of Cal27 and HN6 cells were achieved; the arrows indicated pyroptotic cells. (n = 4). (E–H). Representative bright-field microscopy images and pyroptosis index of Cal27 and HN6 cells after different treatments; the arrows indicated pyroptotic cells. (n = 4, scale bars=200 μm). CG, cathepsin G, MMP9, matrix metalloproteinase 9; NE, neutrophil elastase; NET, neutrophil extracellular trap; OSCC, oral squamous cell carcinoma.


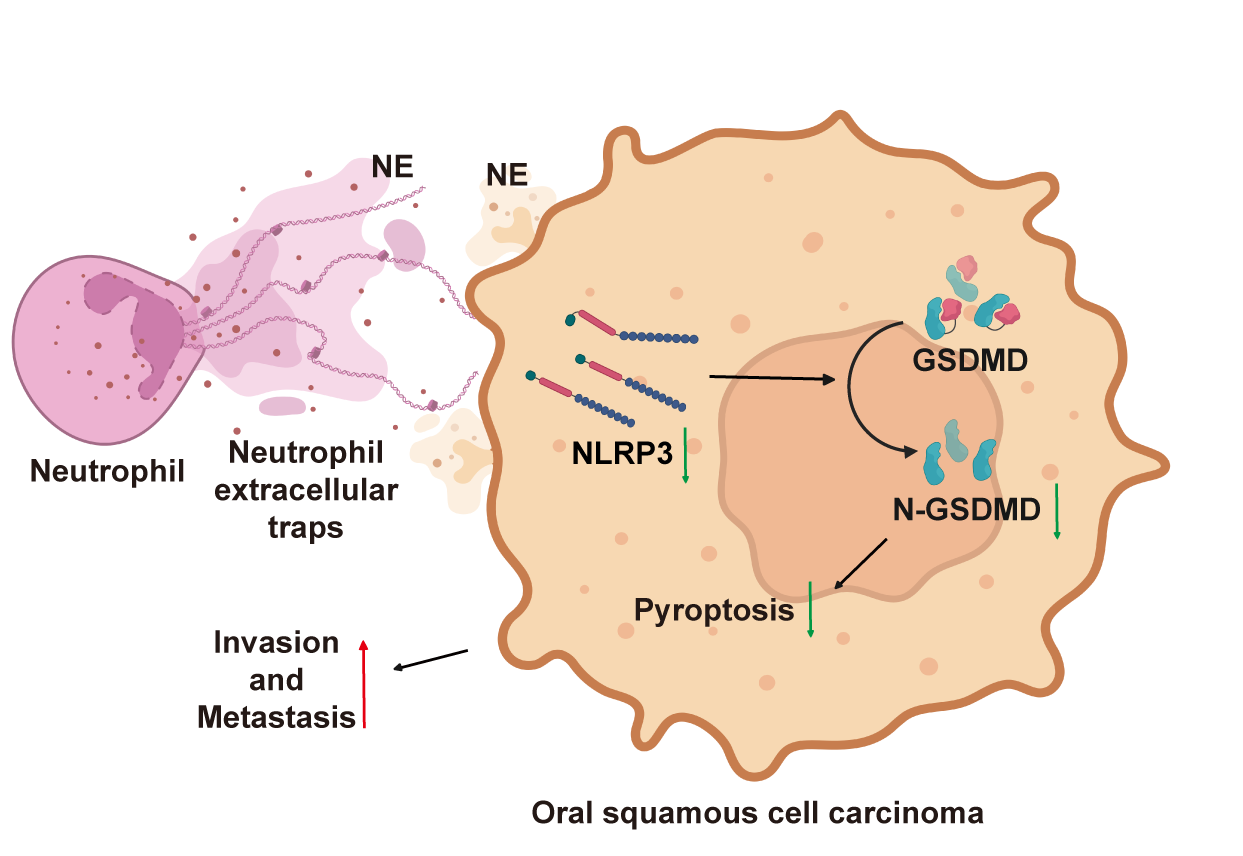


**Figure S9. Schematic diagram highlighting the NE in NETs promotes invasion and metastasis via NLRP3-mediated oral squamous cell carcinoma pyroptosis inhibition.**
