# Supplementary material for: Neutrophil extracellular traps promote invasion and metastasis via NLRP3-mediated oral squamous cell carcinoma pyroptosis inhibition
Source: Cell Death Discov. 2024 May 2;10:214. doi: 10.1038/s41420-024-01982-9 (PMC11066066; doi:10.1038/s41420-024-01982-9)

Figure 3C-Cal27

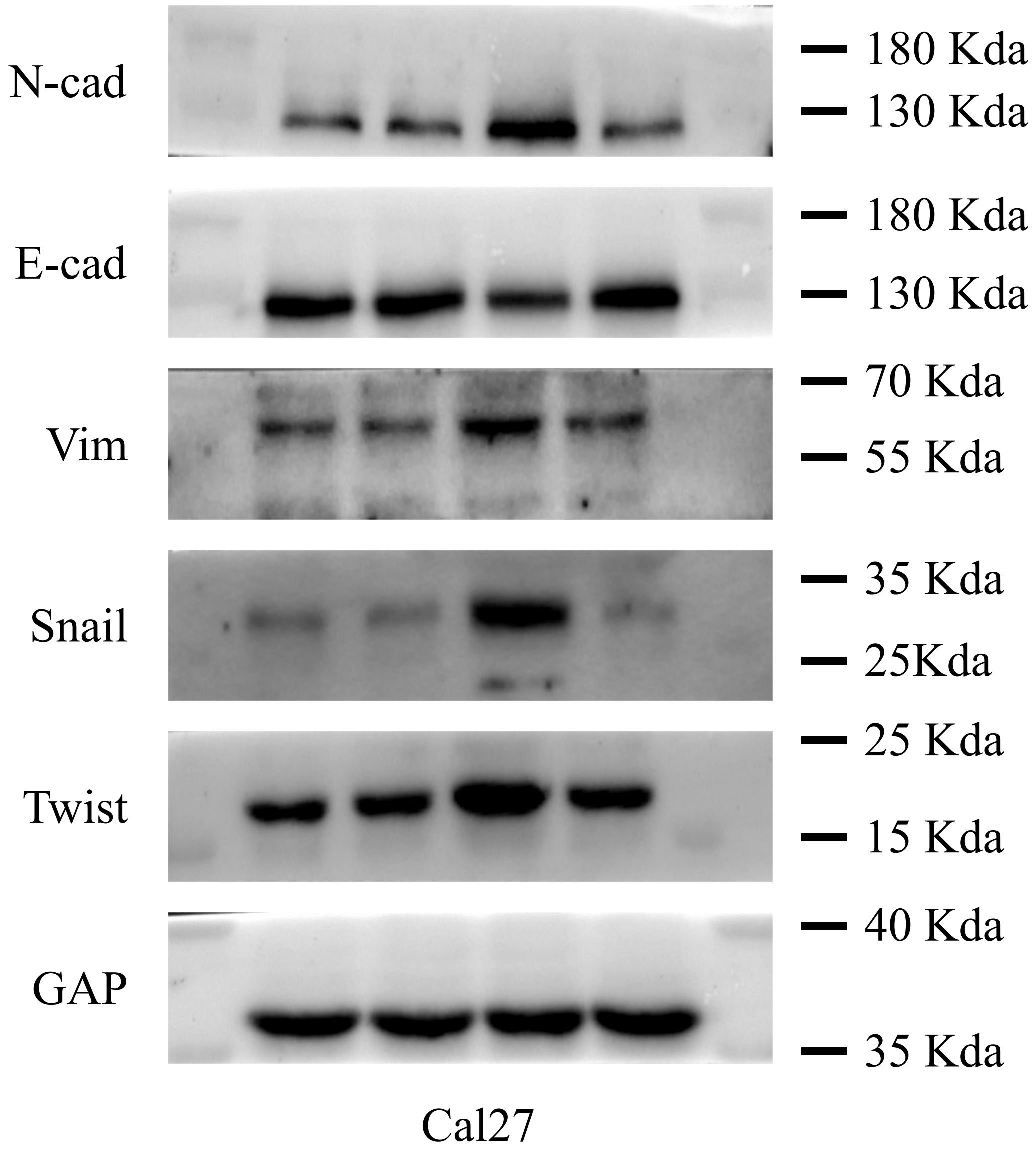

Figure 3C-HN6

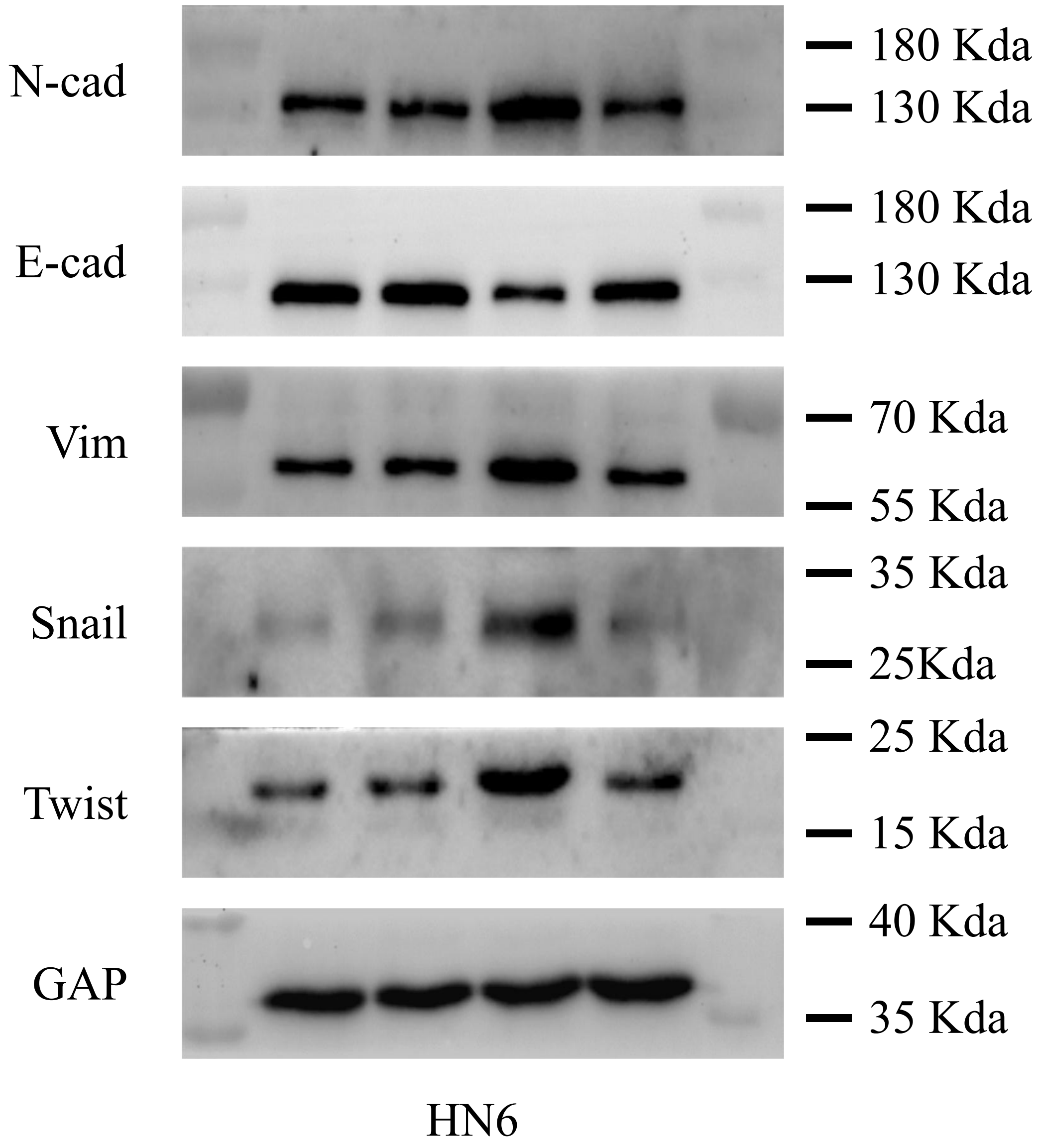

Figure 5C

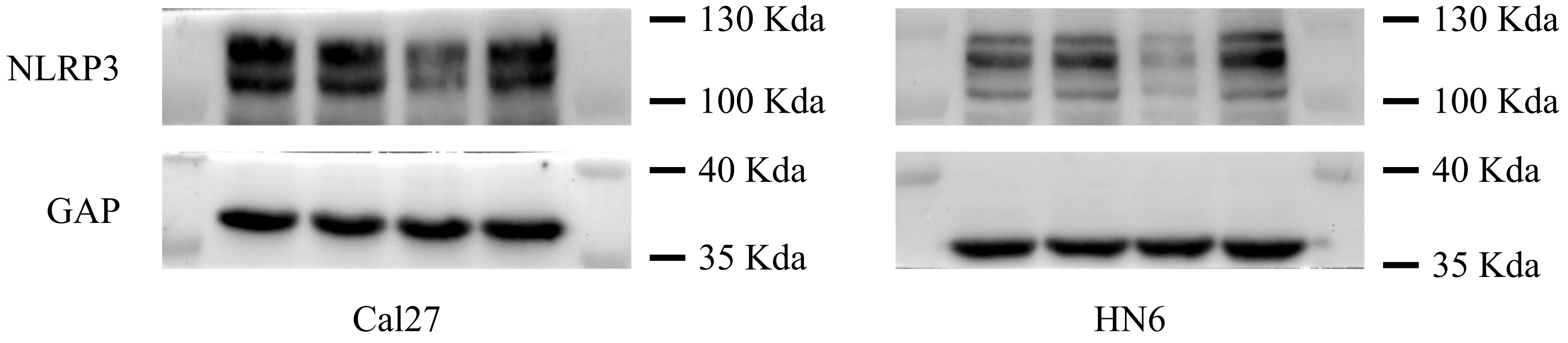
NLRP3
GAP
130 Kda
100 Kda
40 Kda
35 Kda
Cal27
130 Kda
100 Kda
40 Kda
35 Kda
HN6

Figure 5D

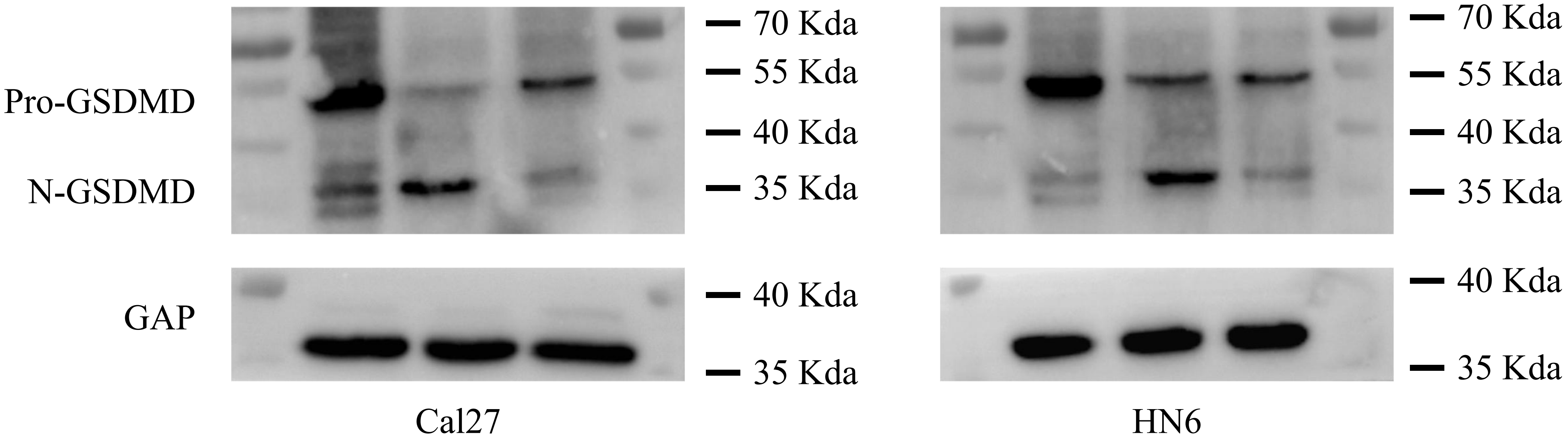
Pro-GSDMD
N-GSDMD
GAP
70 Kda
55 Kda
40 Kda
35 Kda
40 Kda
35 Kda
Cal27
70 Kda
55 Kda
40 Kda
35 Kda
40 Kda
35 Kda
HN6

Figure 6A-Cal27

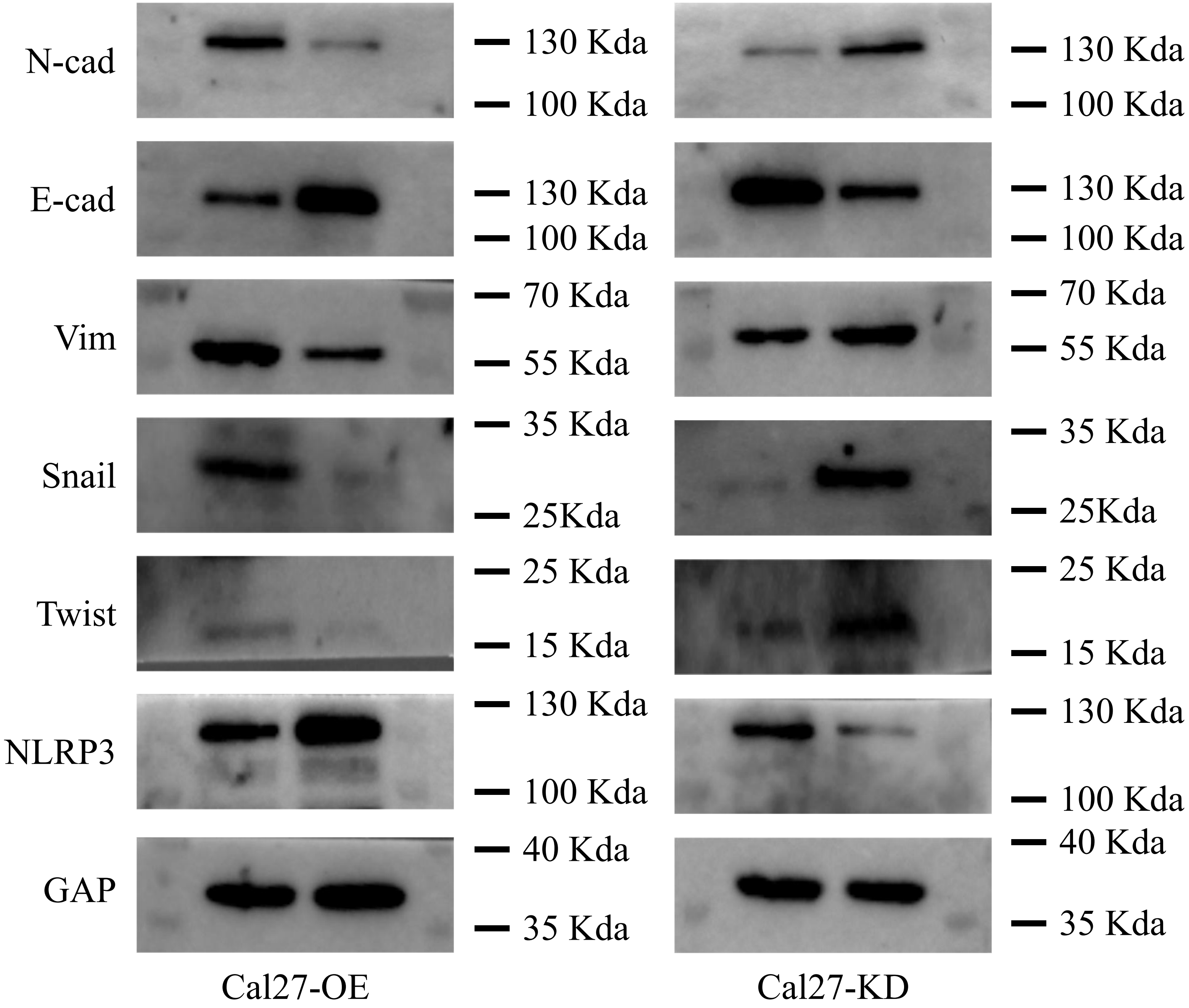

Figure 6A-HN6

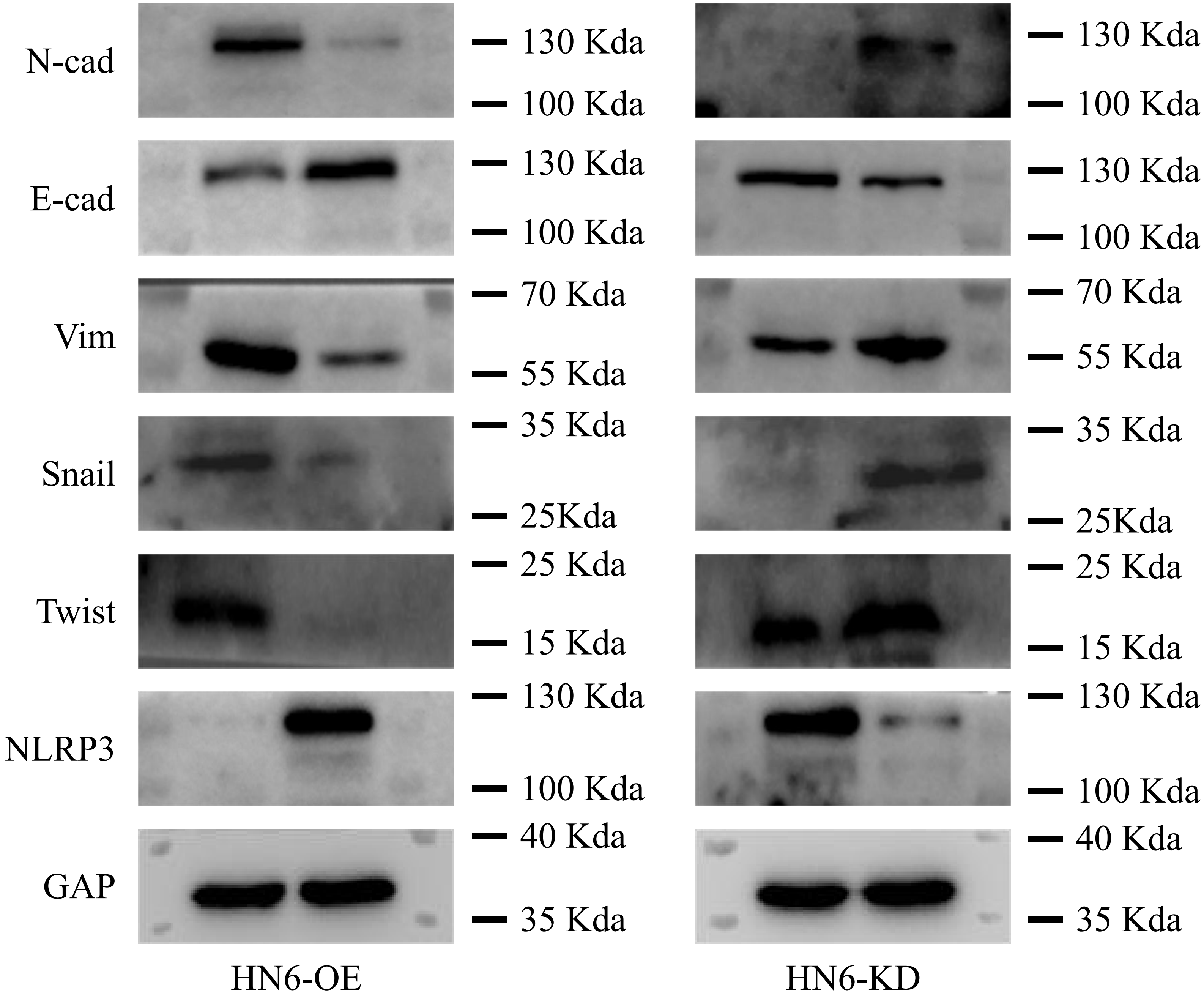

Figure 8A-Cal27

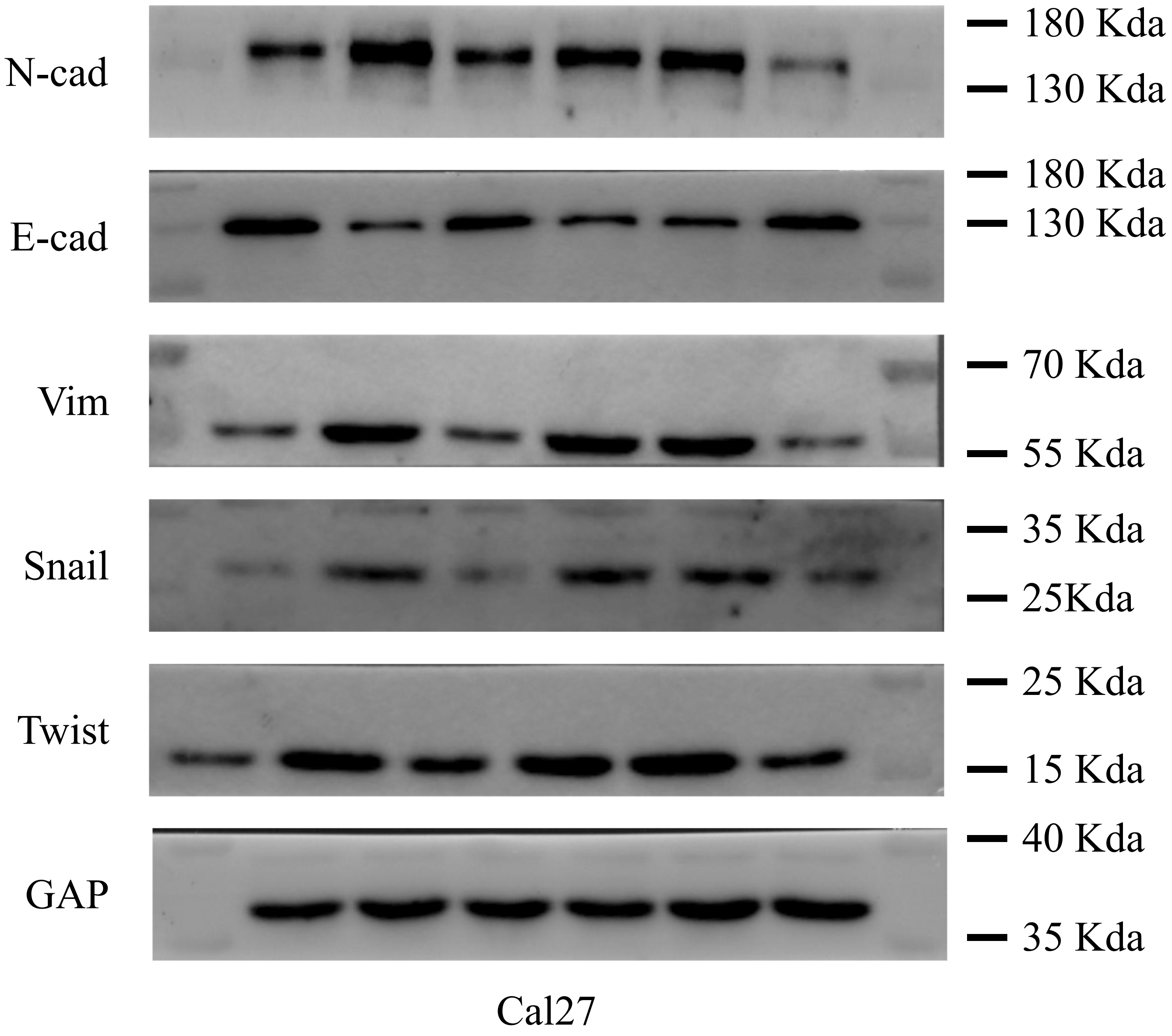

Figure 8A-HN6

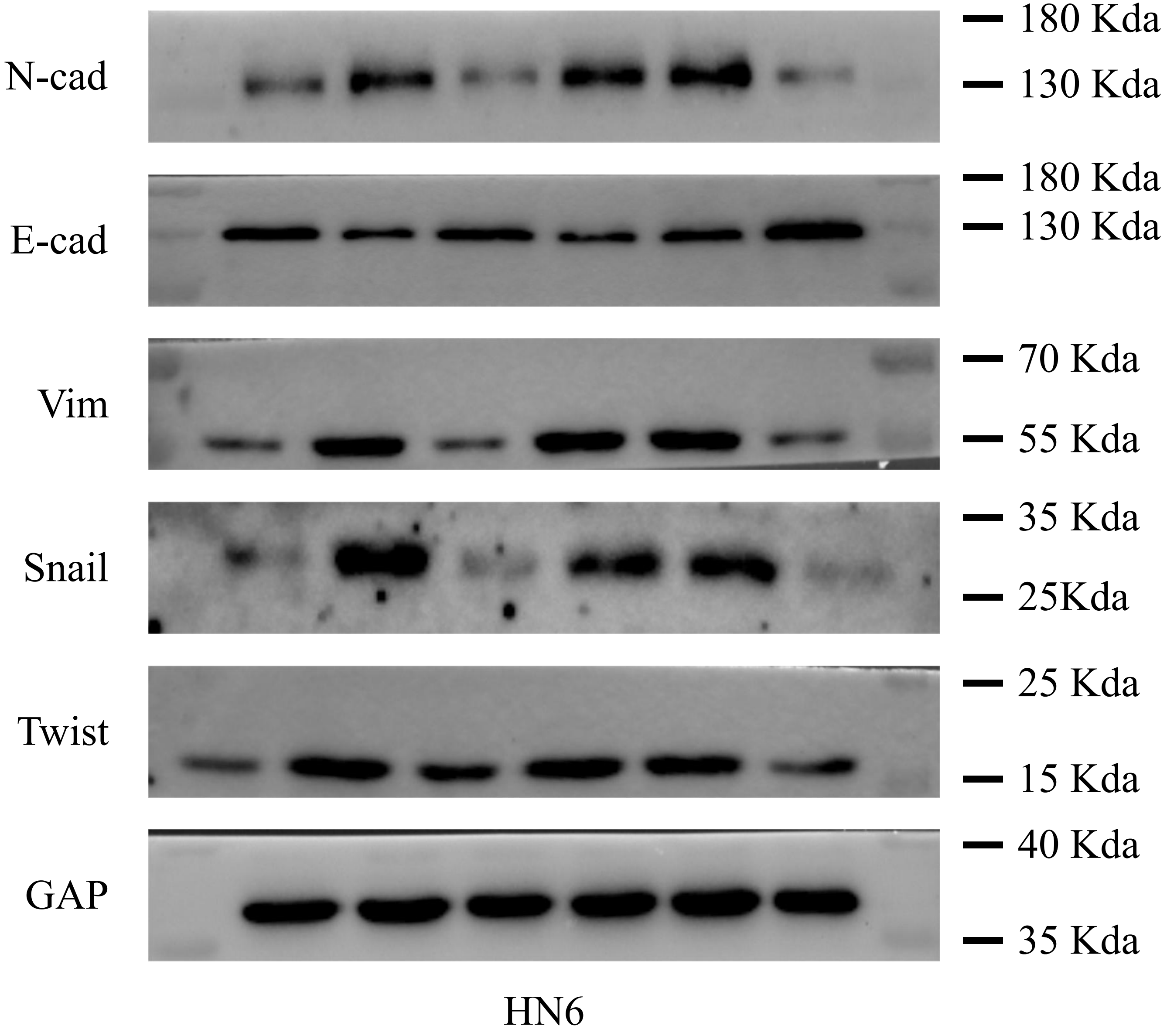

Figure 8B-Cal27

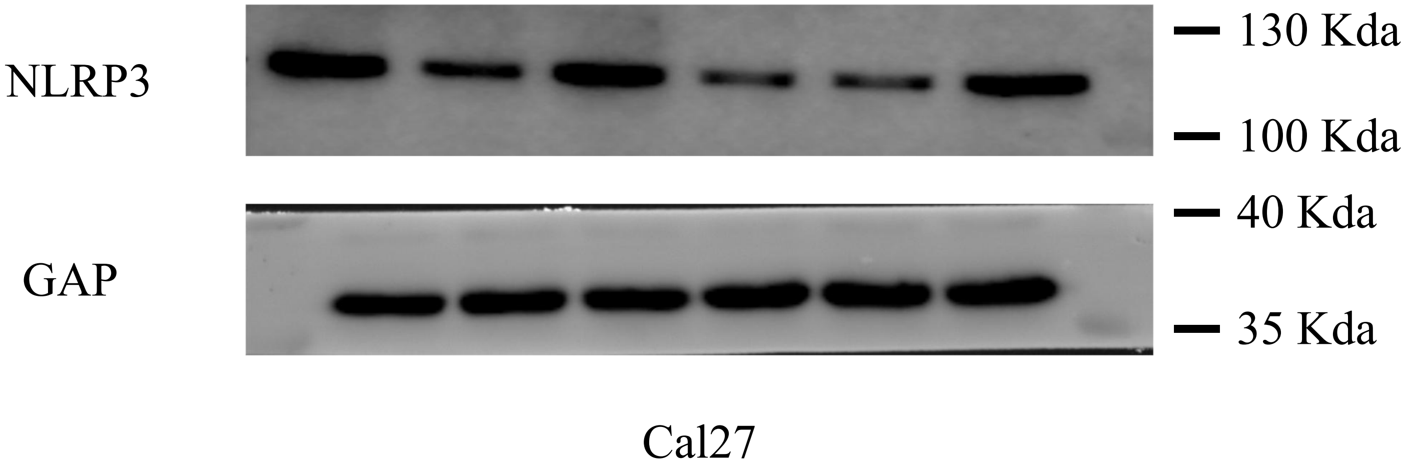

Figure 8B-HN6

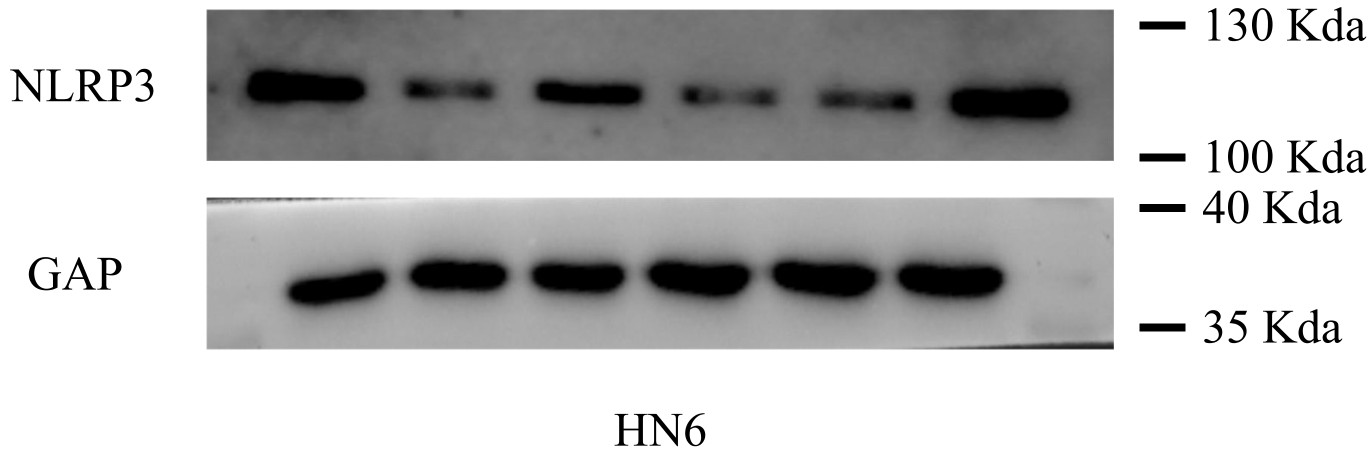

Supplementary Figure 3-Cal27

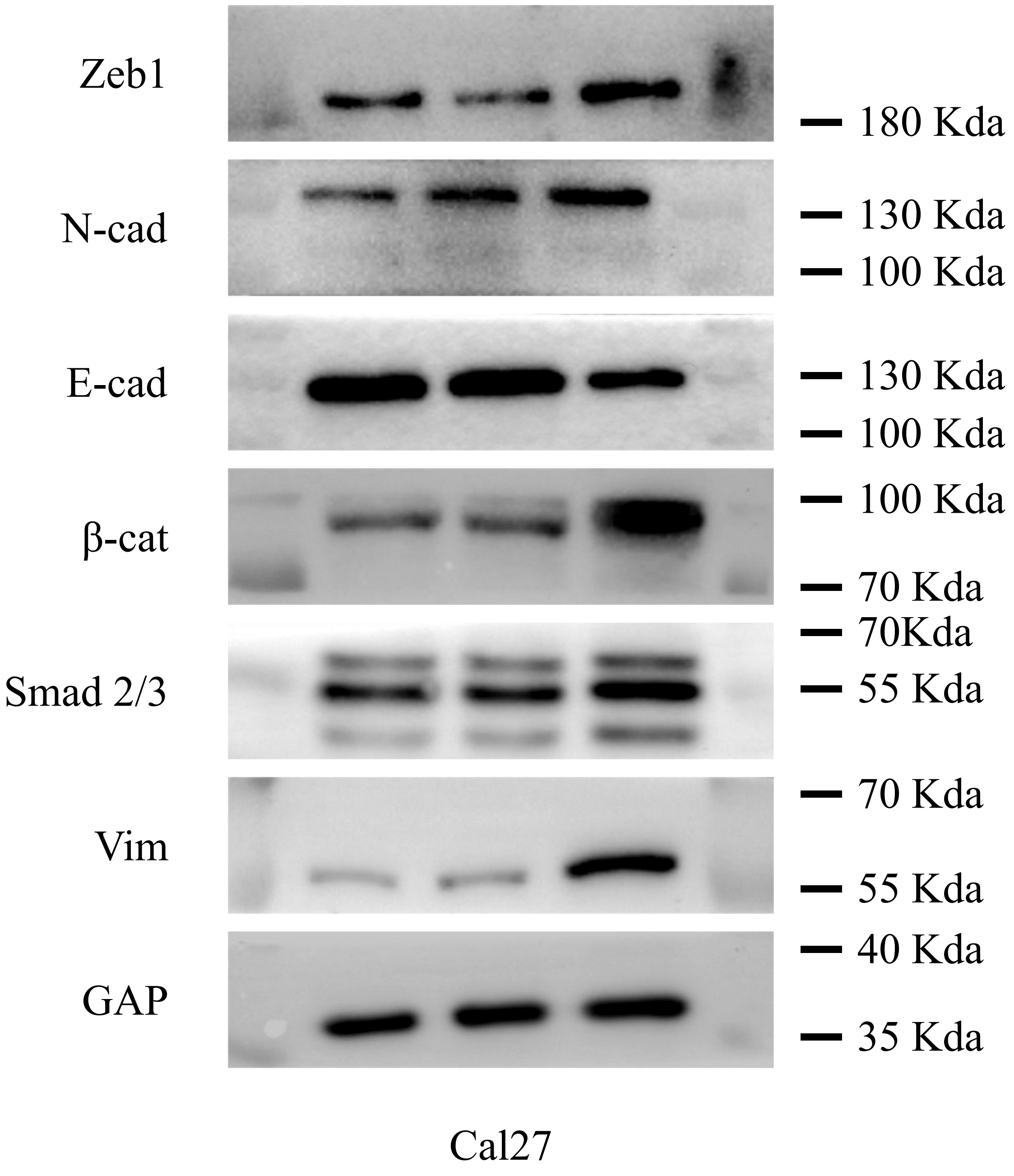

Supplementary Figure 3-HN6

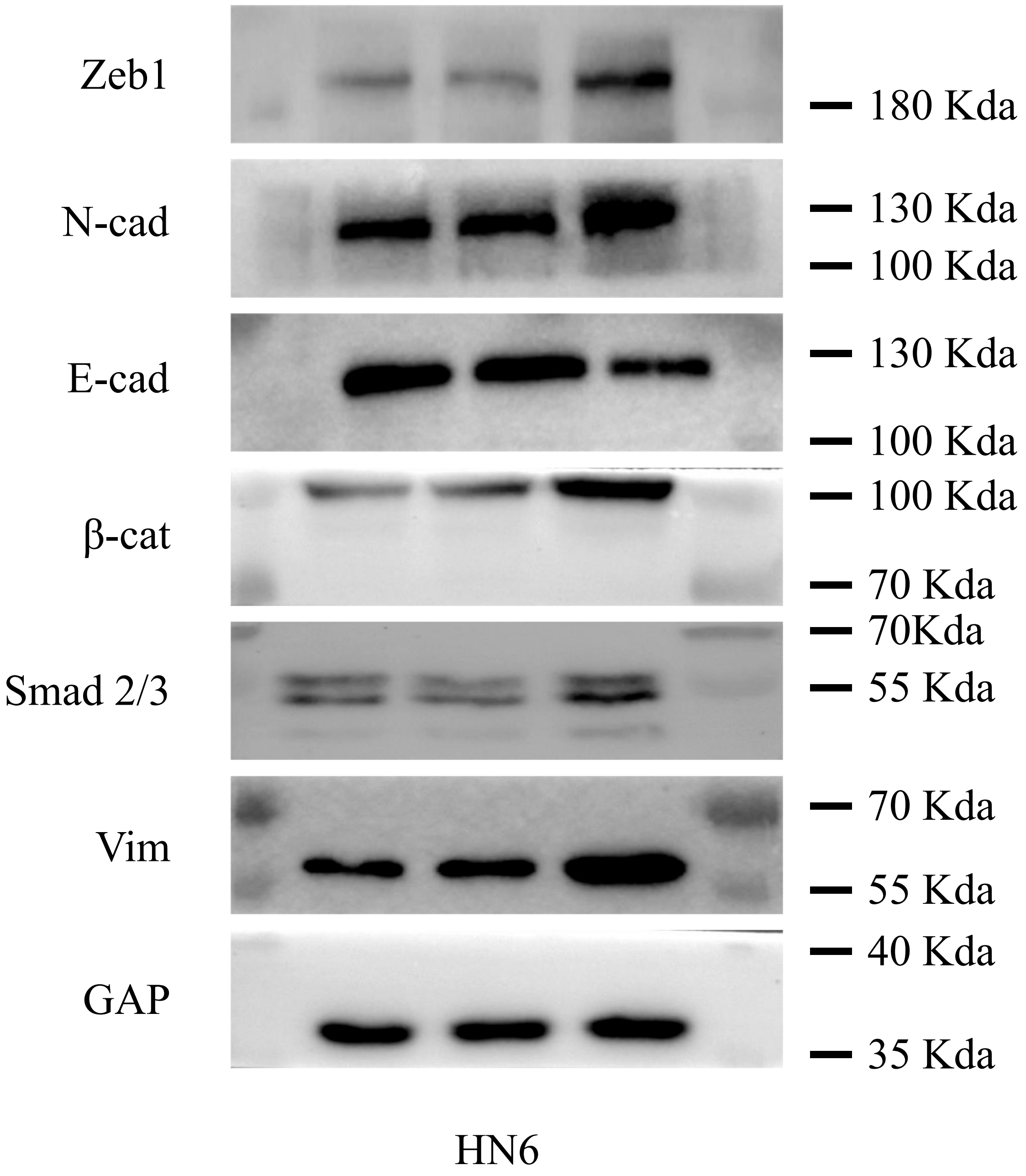

Supplementary Figure 3-Fadu

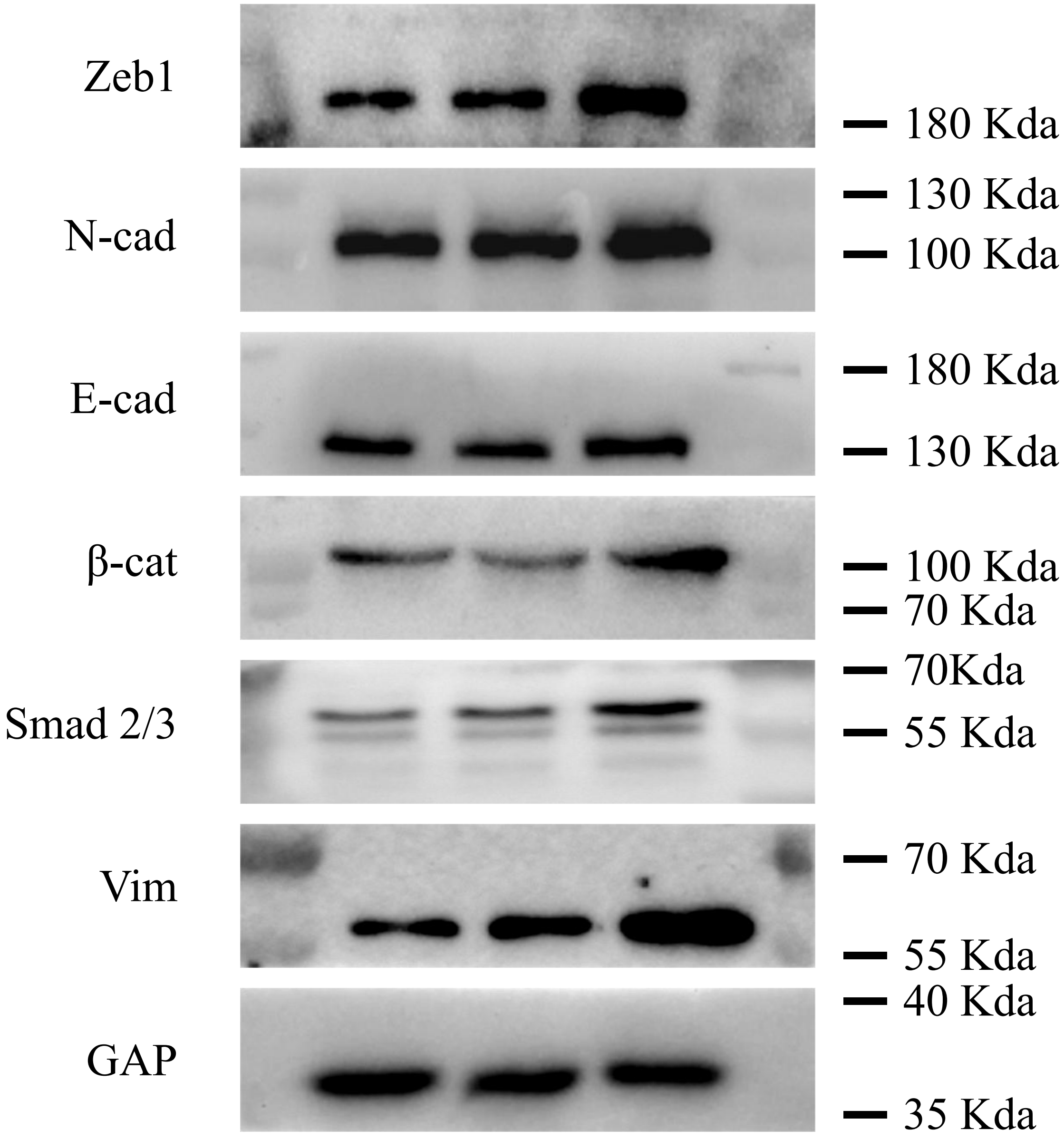

Fadu

Supplementary Figure 3-HN4

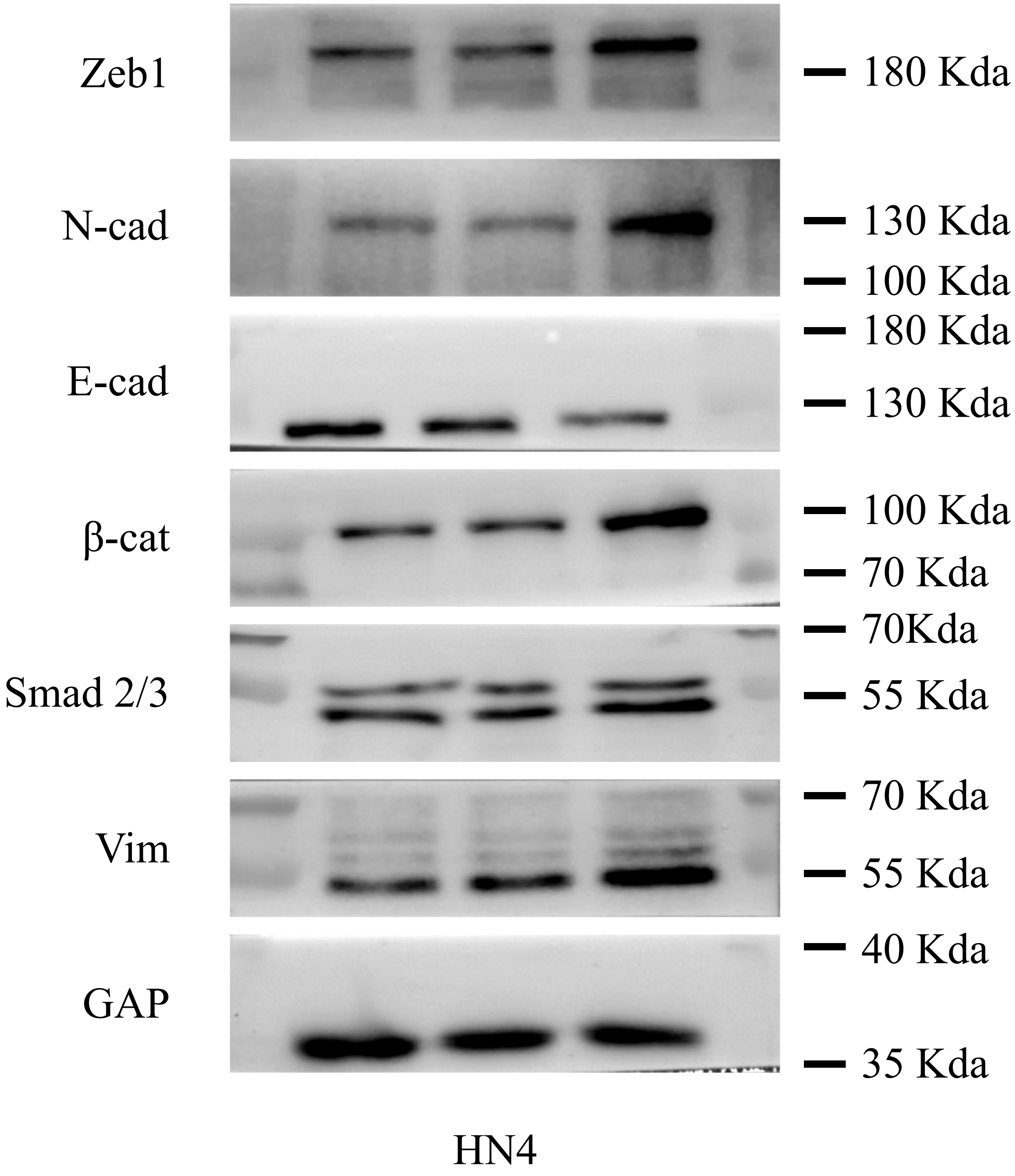

Supplementary Figure 3-HN30

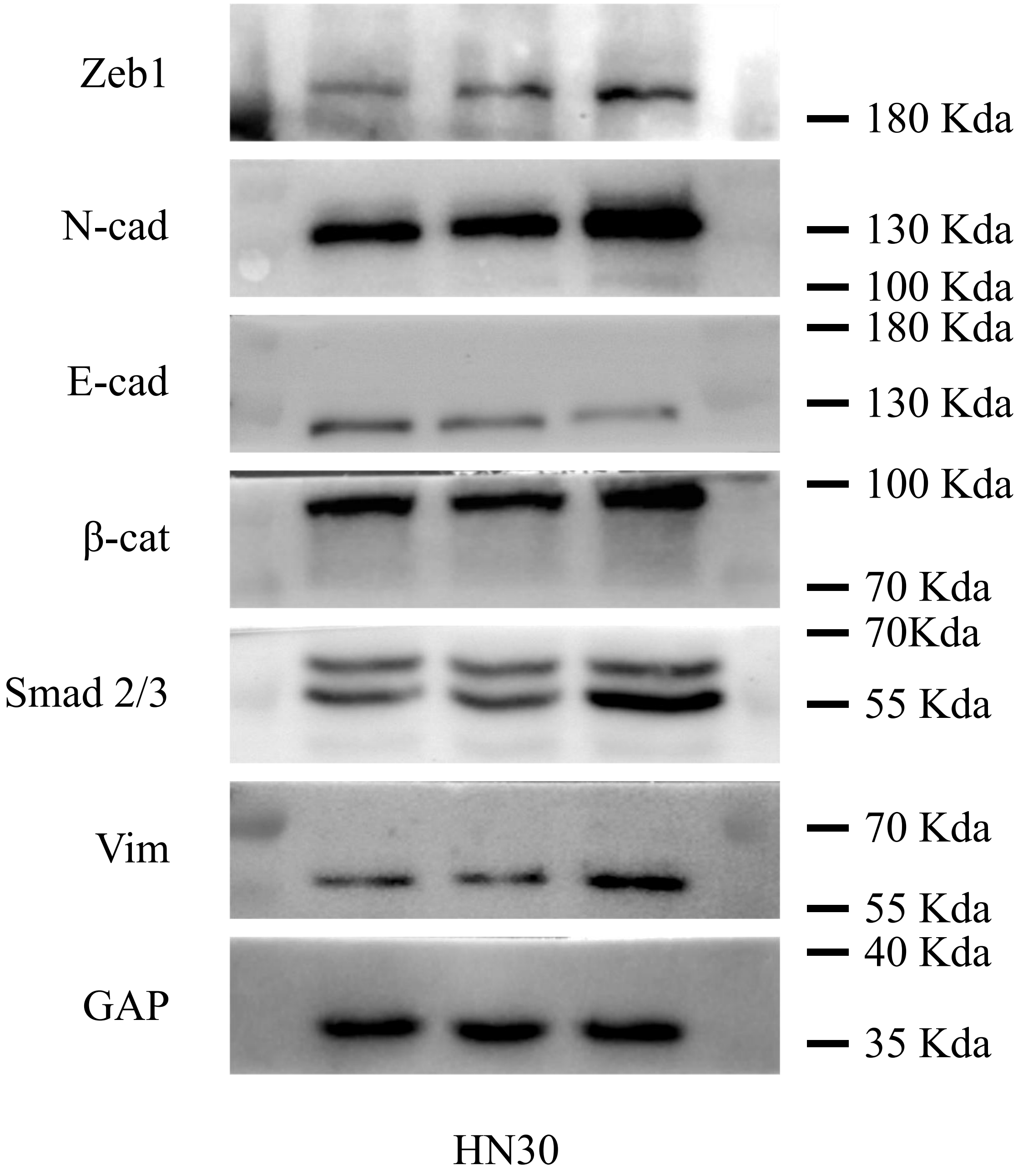

Supplementary Figure 3-HSC3

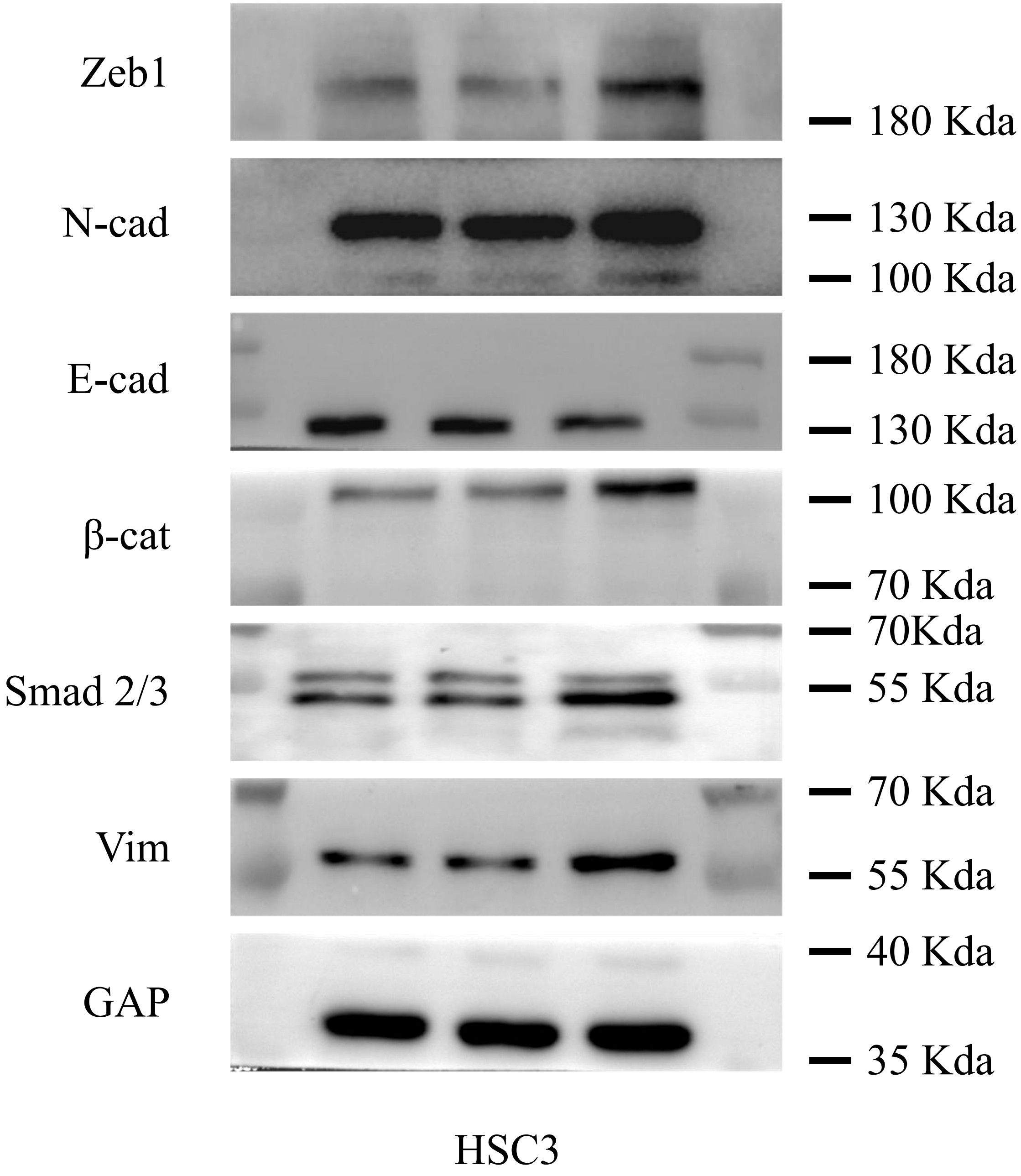

Supplementary Figure 3-SCC4

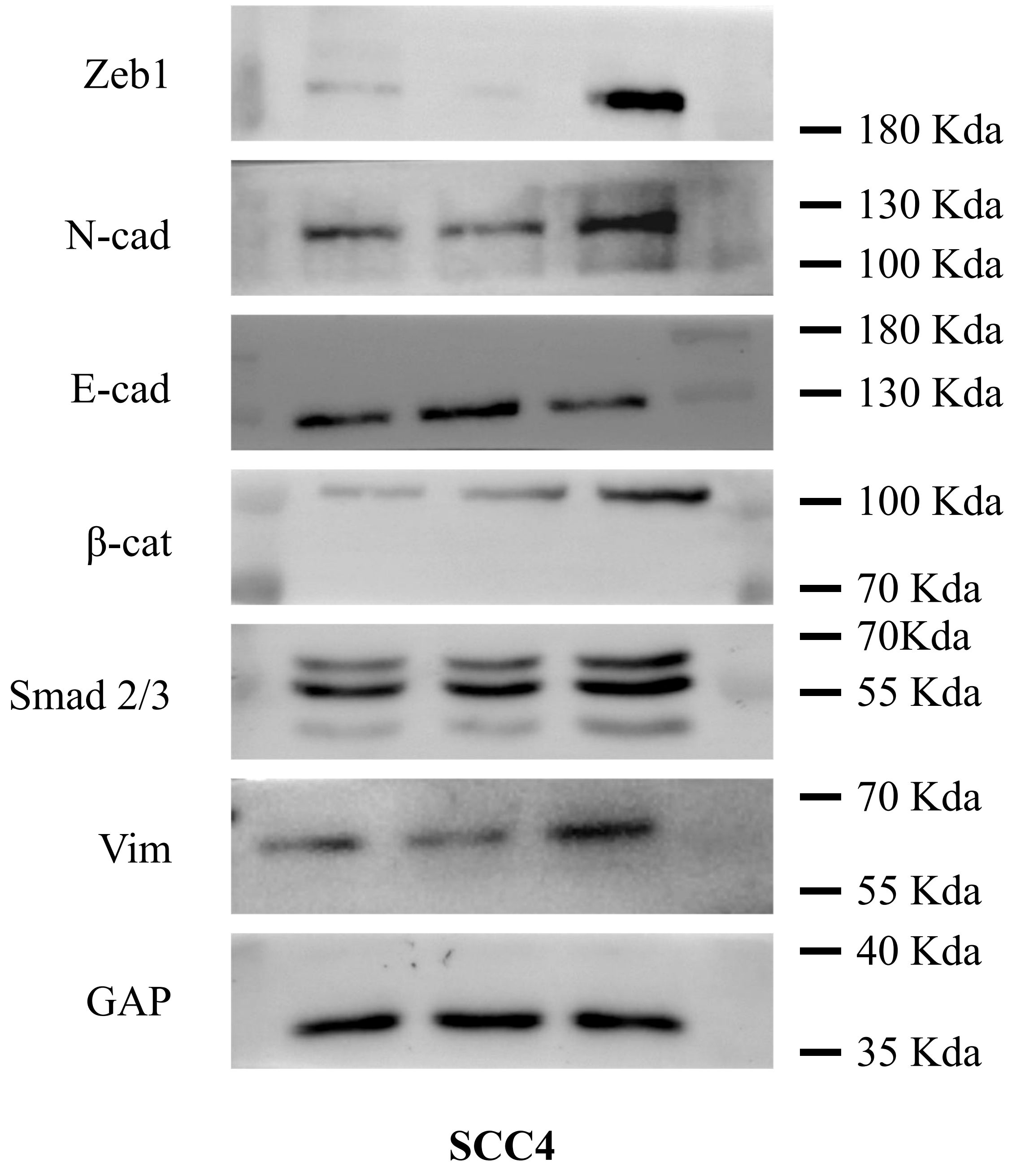

Supplementary Figure 3-SCC9

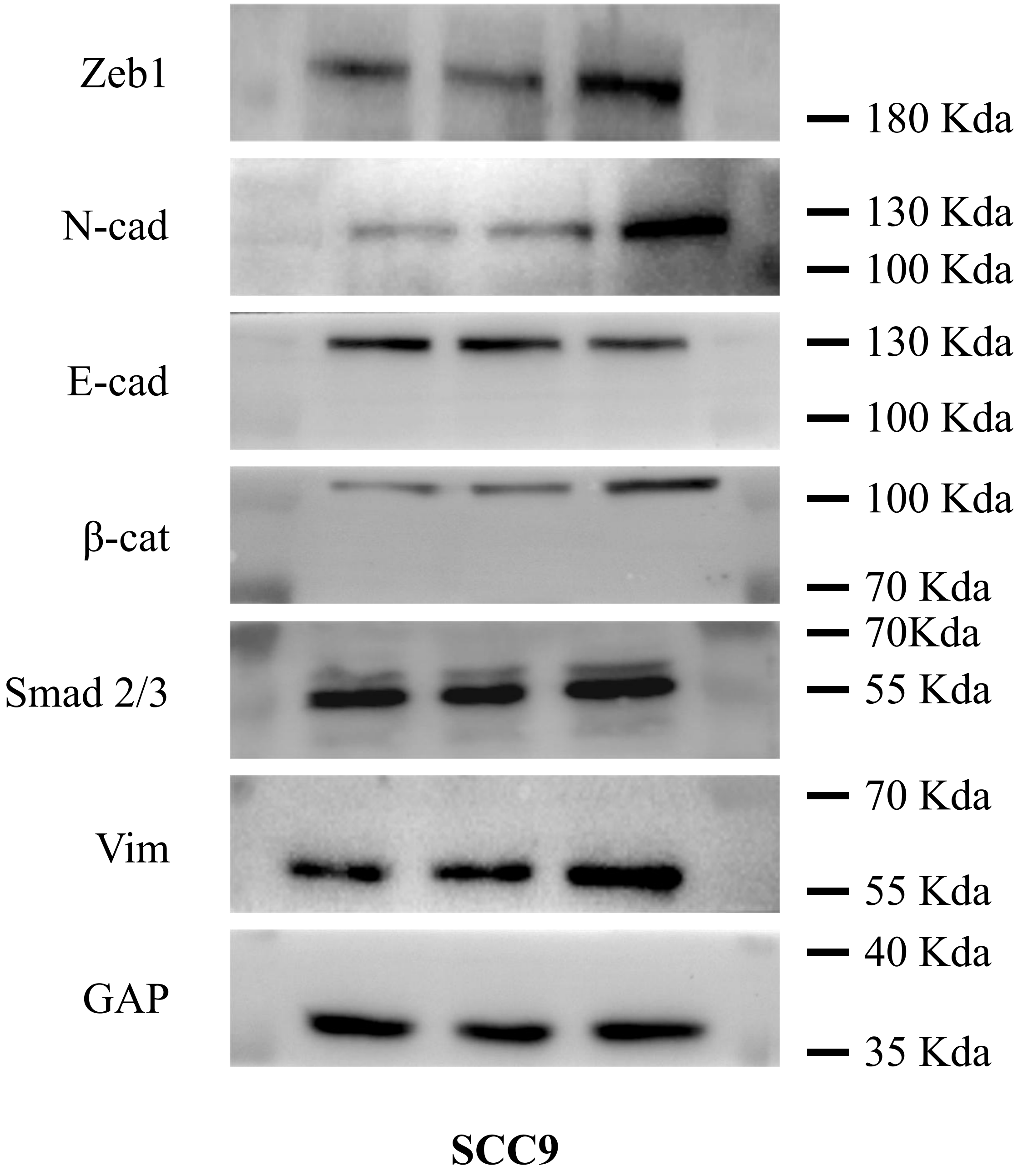

Supplementary Figure 3-SCC25

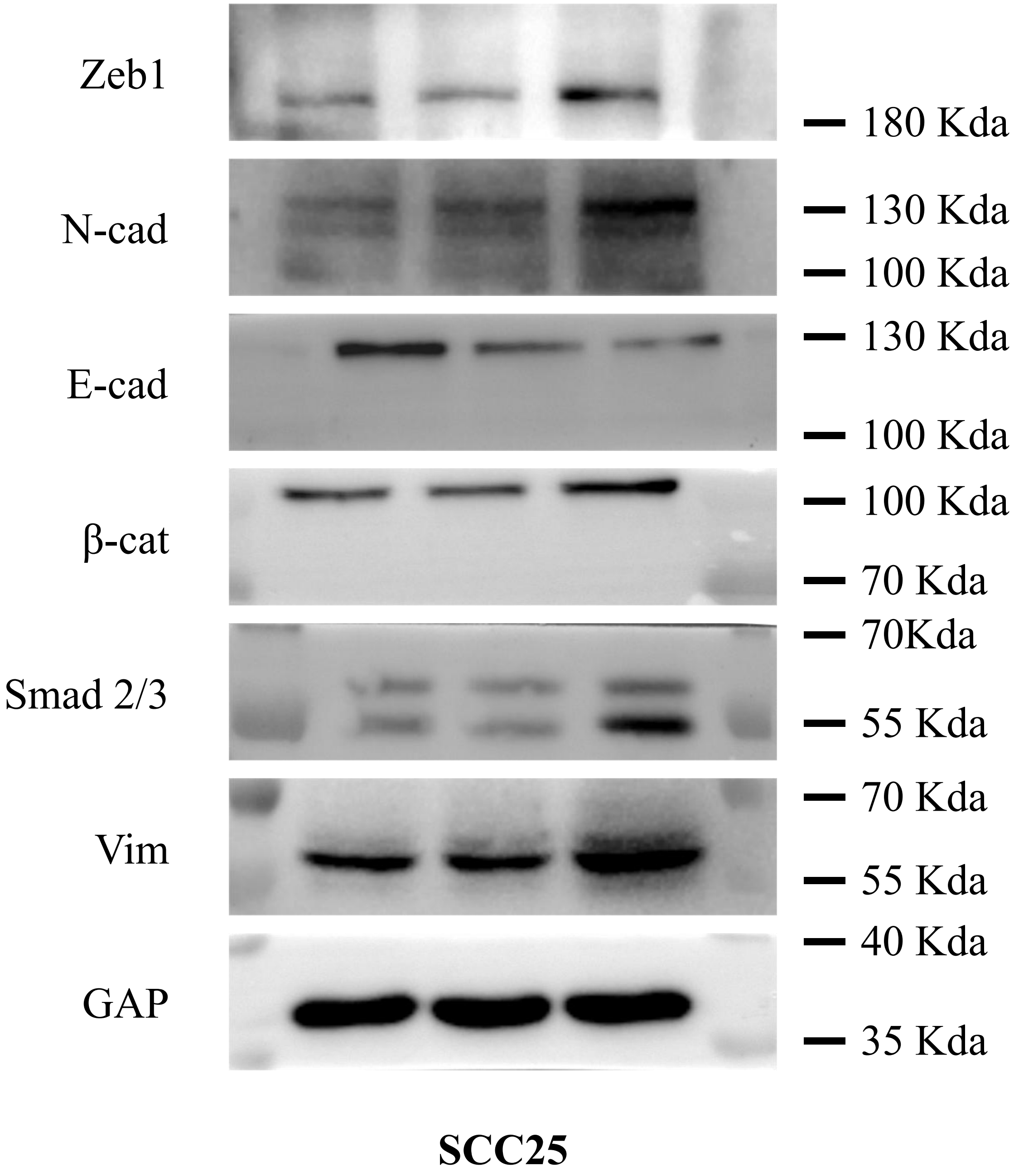

Supplementary Figure 5A

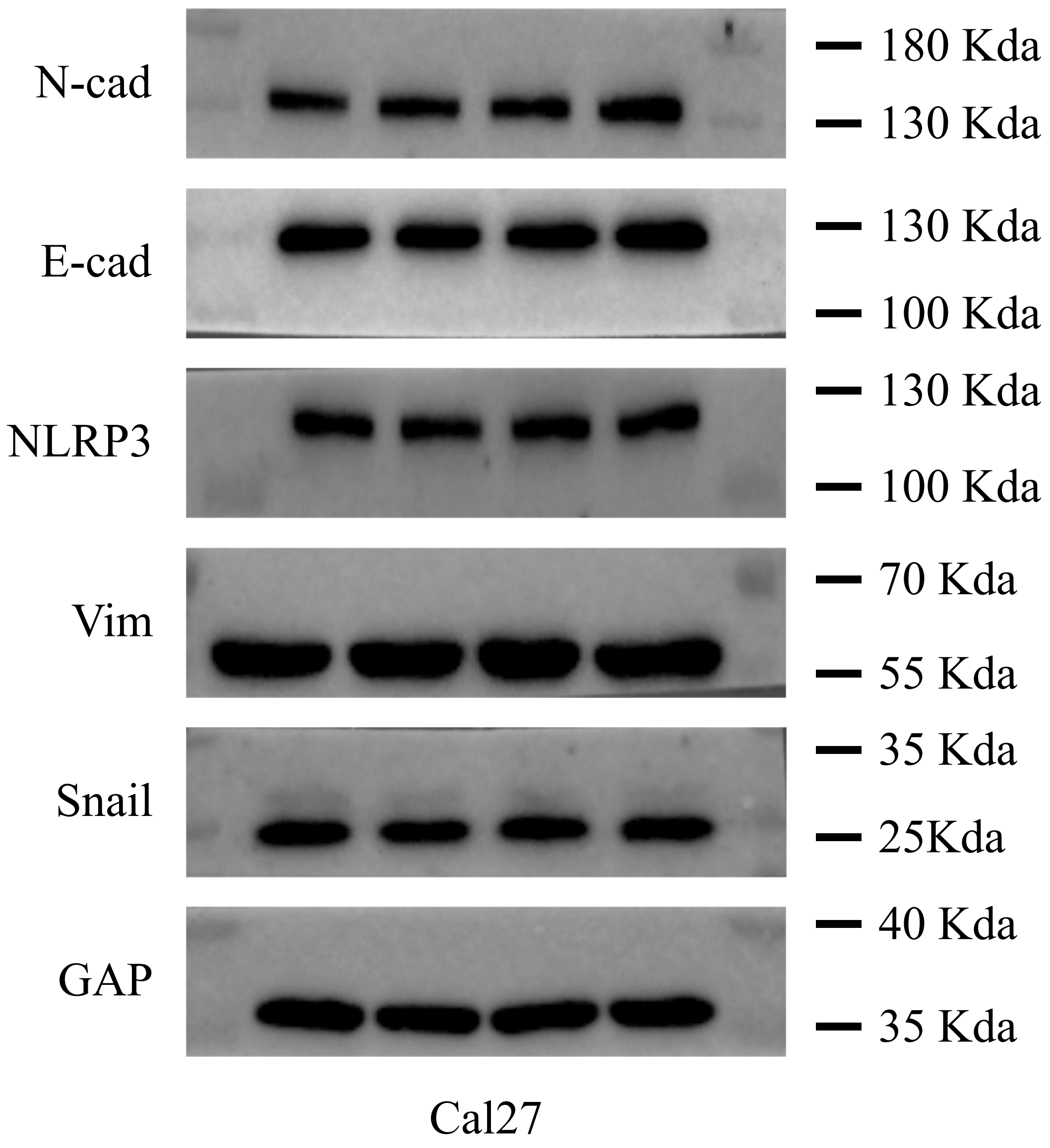

Supplementary Figure 5B

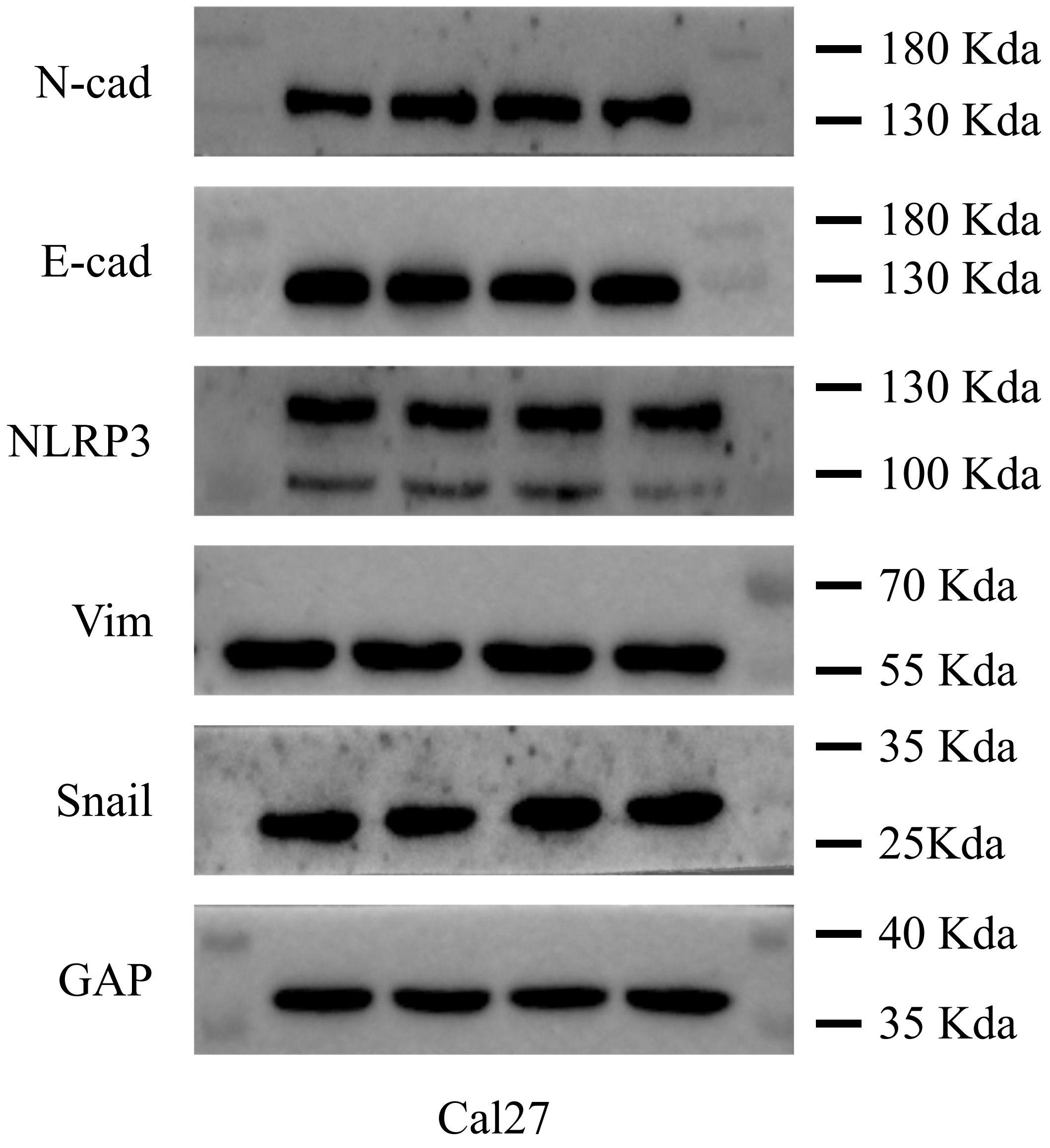

Supplement: Supplementary file 2 — original western blots [file 41420_2024_1982_MOESM2_ESM.pdf]
